# Positive and unlabeled learning from hospital administrative data: a novel approach to identify sepsis cases

**DOI:** 10.1007/s10729-025-09733-7

**Published:** 2025-10-28

**Authors:** Justus Vogel, Johannes Cordier

**Affiliations:** Chair of Health Economics, Policy and Management, School of Medicine, University of St. Gallen, St.-Jakob-Strasse 21, CH-9000 St. Gallen, Switzerland

**Keywords:** Applied data science, Hospital administrative data, Machine learning, Positive and unlabeled learning, Real-world data, Sepsis Detections

## Abstract

In positive and unlabeled (PU) learning problems, only positive examples are labeled. Unlabeled data contain both positive and negative examples. Studies show that positive examples of (secondary) diagnoses, and clinical conditions, such as sepsis, are present in unlabeled hospital administrative data, potentially distorting hospital reimbursement systems, and negatively affecting hospitals’ revenue and profitability. We investigate whether PU learning is suitable for improving the quality of hospital administrative data. We train three models on 313,434 hospital cases using hospital cost features: two based on the two-step “spy” approach and one using a robust PU learning method. For model evaluation, we rely exclusively on positive examples due to the PU setting. To further assess model performance, we perform an external validity check: We relabel unlabeled sepsis cases, derive new sepsis rates, and compare them to those reported in medical record review studies. All models identify true positives well in unseen data. External validity checks show, however, that only the robust PU learner effectively discriminates between positives and negatives in the unlabeled data, yielding new sepsis rates within the range of sepsis rates reported in medical record review studies. PU learning can improve the quality of hospital administrative data, but its effectiveness depends strongly on the choice of learning approach and classifier. The output of a PU learner can potentially improve hospital reimbursement systems, hospital revenue and profitability management, and sensitivity analyses in healthcare management science, health economics, health services research, and disease surveillance.

**Supplementary Information:**

The online version contains supplementary material available at 10.1007/s10729-025-09733-7.

## Highlights

- We apply positive and unlabeled (PU) learners to identify and relabel sepsis cases in unlabeled hospital administrative data
- By employing different classification thresholds for relabeling unlabeled sepsis cases, we simulate new sepsis rates, evaluating model results
- Evaluation shows that one model can effectively identify positive sepsis cases part of the unlabeled data
- Hospital management, reimbursement regulators, and researchers can use PU learners to increase the quality of hospital administrative data
- PU learner output could be used to improve hospital reimbursement systems, hospital revenue and profitability management, and research

## Introduction

Commonly, binary classifiers are learned from a fully labeled training set. In practice, situations arise, however, where only one class, usually positive examples, is labeled and the unlabeled data contain both negative and positive examples. Accordingly, the subject of learning from positive and unlabeled (PU) data has received increasing attention in the machine learning research community in the last two decades [1–5].

Large hospital administrative or billing datasets are prone to limitations regarding the documentation as well as correct and consistent coding of certain (adverse) events [6]. Only positive instances of e.g., secondary diagnoses are actively coded and uncoded instances are assumed to be negative rather than unlabeled. This issue is well-researched for the coding of sepsis cases, commonly under- and/ or misreported in hospital administrative data [7–11]. Studies rely on medical record review by experts checking whether the absence of a positive label for sepsis diagnosis codes actually means that the patient did not suffer from sepsis. Following this approach, Mellhammer et al. [8] find, for instance, that inpatient sepsis rates estimated from administrative data were 1.0% to 1.4% while medical record review revealed a sepsis rate of 4.1%. Similarly, Schwarzkopf et al. [7] report low sensitivity (26.8% to 38.0% depending on sepsis definition) for sepsis detection via diagnosis codes from hospital administrative data. Using medical record review, the authors found that in their sample of more than 10,000 cases from ten German hospitals, incidence of severe sepsis-1 was at 3.3% instead of 1.4% as estimated from hospital administrative data.

Such PU data-related issues could affect hospital reimbursement systems relying on administrative data, e.g., diagnosis-related group (DRG) systems as used in Switzerland and Germany [12, 13]. DRGs are grouped, among other information, using diagnosis and procedure codes. As for both negative examples could rather be regarded as unlabeled containing both positive and negative examples, DRG grouping might be distorted. For instance, positive and negative sepsis cases part of the unlabeled data might be grouped together. Consequentially, resource-intensive, costly sepsis cases are part of the same DRG as less complex, less costly cases. DRG flat rate payments are calculated from cost data to represent the average treatment costs incurred by a typical hospital. On DRG system level, PU data-related issues can trigger that the calculated DRG cost weights are based on cases with a very different resource consumption, resulting in unaccounted cost variation and non-representative cost weights. For hospitals, the economic consequences of mislabeling a sepsis case (or any other diagnosis or procedure) can be severe. While incurring treatment costs for a complex case, the DRG flat rate payment will only cover the treatment costs of a less complex case, likely to result in a negative contribution margin. For research, values of quality indicators used in health economic and health services research, for instance inpatient complication rates or patient safety indicators, might be higher or lower than those that are currently estimated from administrative data [14]. Similarly, epidemiological studies for disease monitoring or disease burden estimation, e.g., [15–17], might misstate prevalence derived from hospital administrative data [10].

Studies cannot use laborious, costly methods such as medical record review to correctly label tens of thousands of observations or even more, e.g., in a national database. PU learning models, on the other hand, which have proven effective in comparable applications, might constitute a more efficient approach to increase administrative data quality. PU learning algorithms have been applied in biology and bioinformatics, e.g., for gene prediction and detection [18, 19] and gene network inference [20], in text classification [21, 22], and various other applications [23]. While there are some PU learning applications investigating hospital management topics [24, 25], to the best of our knowledge, PU learning has so far not been applied to increase the quality of hospital administrative data.

Based on the problematization, research gap, and potential of PU learners, we investigate the following research question: Is PU learning suitable to increase the quality of hospital administrative data?

## Methods

### Data

#### Application example

We focus on sepsis as application example due to three reasons. Firstly, it is well-researched that sepsis is commonly underreported when only relying on hospital administrative data. While there is evidence that the reporting of sepsis in hospital administrative and claims data has increased in the last two decades (e.g., [11, 26]), the above cited, very recent studies indicate that the issue persists [7, 8]. Thus, we may reasonably suspect that there is a considerable amount of positive sepsis cases in the unlabeled data. Secondly, sepsis is a major complication for both surgical and non-surgical patients [27]. This underscores its medical relevance and is advantageous regarding data volume PU learners can learn from. Thirdly, once afflicted, sepsis patients’ health status deteriorates considerably, sepsis is strongly linked to high inpatient mortality, and its economic burden is considerable [17, 26, 27].

#### Learning attributes and data source

We hypothesize that a classifier might learn positive examples of sepsis cases from hospital cost data. Costs accrue from treatment. While sepsis might not have been documented by physicians and/ or nurses and/ or it might not have been coded from documented information by coding specialists (or in fact it might not have been diagnosed at all) [7, 26], we assume that symptoms are observed and treated. While a non-treatment might occur in single instances, it should not occur systematically. Indeed, a deliberate non-treatment of symptoms would be highly unethical. Consequentially, positive examples of sepsis part of the unlabeled data should exhibit similar cost patterns as actual positive examples.

We exploit the Hospital Case Cost Statistic from the Swiss Federal Statistics Office for data years 2017 to 2019. This dataset should be well-suited for PU data classifiers to learn from hospital cost data as it contains 71 distinct cost attributes and additionally total costs at case level. There are eleven variable, direct cost categories including, for instance, pharmaceuticals, blood and blood products, medical materials, and implants. The remaining 60 cost attributes refer to indirect costs. More specifically, in the dataset, 30 service centers are listed, comprising medical services such as intensive care unit (ICU) physician services, pathology, physiotherapy, dialysis, laboratory analyses, anesthesiology, the operating room area, emergency services, and diagnostic imaging, as well as tertiary services such as patient administration and hospitality services. For each of these service centers, two indirect cost categories are given, namely one including indirect costs without asset usage costs and one including only asset usage costs.

Besides, the dataset contains information on cases’ gender, age (5-year age bands), admission reason, discharge reason, main treating hospital department, diagnosis-related group code, the main diagnosis, up to 49 secondary diagnoses, the main procedure, up to 99 additional procedures, and many other patient characteristics. We deliberately do not use these attributes for learning our PU learner due to two reasons: Firstly, patient characteristics such as age, gender, and co-morbidities might be well-suited to predict the risk of developing sepsis. For risk prediction, a fully labeled dataset and supervised learning methods would be used. In PU learning, the setting is distinctly different, however. In addition, further analysis with PU learner outputs might be biased if the same attributes were used in the PU learning process that would later also be used for, e.g., risk-adjustment of outcome indicators or DRG grouping. Secondly, attributes such as the DRG code, specific procedures, and the treating hospital department should be well-reflected in the cost attributes. For learning our PU classifier, we thus focus on cost attributes.

There are several tens of thousands of ICD-10-codes and procedure codes. Each case receives one main diagnosis and between one and 100 procedures. The number of possible combinations of main diagnosis and procedures is vast. It is perceivable that there are combinations that lead to similar cost patterns as seen for labeled and unlabeled positive sepsis cases. Thus, we complement cost attributes with aggregated information on main diagnosis and performed treatments (see Table 6 in the supplements for a full list) to enable our PU learner to efficiently learn sepsis cases from cost data.

Lastly, the Hospital Case Cost Statistic includes all acute somatic care cases hospitalized with complete data for the calculation of DRGs from Swiss hospitals that report such data [13]. Compared with the Swiss Medical Statistic, which includes all acute somatic care cases irrespective of data completeness or reporting obligations, the Hospital Case Cost Statistic contains about 24% hospitals and 38% cases less for our observation period. We argue that exclusion of cases in the Hospital Case Cost Statistic is independent of the labelling mechanism for sepsis cases, however: Labelling occurs shortly after patient discharge and is performed by medical coding specialists of a hospital while data cleaning is performed by the SwissDRG AG, the organization responsible for DRG development and revision in Switzerland, and at a later point in time. Moreover, non-participation of a hospital in cost data reporting, e.g., because it does not have a public service mandate, is also independent of sepsis case labelling.

#### Identification of sepsis cases

Commonly, sepsis cases are identified in hospital administrative data via diagnosis codes specified in the International Classification of Diseases, Version 10 (ICD-10). Yet there is no one gold standard what diagnosis codes or combination of codes to use for the identification of sepsis cases [27]. Usually, authors rely on different explicit and implicit coding strategies. Explicit coding means that ICD-10-codes concretely naming sepsis as clinical condition are used while implicit coding strategies rely on combinations of ICD-10-codes strongly linked to sepsis. We train our PU learner with positive examples according to an explicit coding strategy. To evaluate our PU learner, we use the same explicit as well as an implicit coding strategy (Angus definition [16], i.e., organ dysfunction in combination with an infection), described in more detail below (see Table 5 in the supplements for used ICD-10-codes). Schwarzkopf et al. [7], for instance, also used these coding strategies.

#### Inclusion and exclusion criteria, undersampling and size of final sample

There are roughly 2.9 million inpatient cases available in the Hospital Case Cost Statistic for our observation period. As sepsis can potentially occur in any patient, we include all adult acute somatic care inpatient cases. In our dataset, age is recorded in 5-year age bands (0–4, 5–9, 10–14, 15–19, etc.). To only include adults, we only include cases 20 years of age and older. In addition, to clean the dataset, we exclude cases without main diagnosis or DRG code, missing cost data, zero total costs, and a length of stay of zero days.

Applying the above inclusion and exclusion criteria and data cleaning steps, the sample would still comprise more than 2.6 million cases, however, with an explicit (implicit) coding sepsis rate of roughly 2.40% (6.58%). To balance the dataset and manage computation time, we reduce the number of negative (or rather unlabeled) sepsis cases (undersampling). To this end, we extract all 63,434 observed positive sepsis cases (explicit coding) and add a random sub-sample of 250,000 unlabeled sepsis cases from the initial dataset. Our final sample comprises 313,434 cases with a positive class label mean of 20.24%.

### PU learning

We first briefly formally review binary classification and preliminaries relevant for PU learning (e.g., the labeling mechanism). Second, we discuss the underlying assumptions for PU learning in the context of hospital administrative data. Third, we present the learning techniques we applied. Lastly, we outline our model evaluation strategy including an external validity check, which we will show is indispensable when evaluating PU learning applications with real-world data. Regarding methodology, we rely on the work of Bekker and Davis [2], who have provided a thorough review of the current state of knowledge regarding PU learning.

#### Formal descriptions

PU learning is a type of binary classification. Yet it differs succinctly from (a) (semi-) supervised learning, and (b) learning from positive-only or one-class data. Bekker and Davis [2] outline that the main differences are that (a) only positive examples are reliable and there are no negative examples to learn from and (b) not only positive but also unlabeled examples in the training set are used for learning.

When training a binary classifier to predict the class label of an example, training examples are tuples $(x,y)$, $x=\{{a}_{1}, {a}_{2}, \dots {a}_{k}\}$ describing a vector of attribute values of data instance $x\in X$, and $y\in \{\text{0,1}\}$ denoting the class label ($y=1$ indicates a positive and $y=0$ a negative example). To train a binary classification learner, it is assumed that the training set is an independent and identically distributed (iid) sample of the real distribution:

$${\varvec{x}} \sim f(x)$$

1 $$\sim \boldsymbol{\alpha }{{\varvec{f}}}_{+}\left({\varvec{x}}\right)+(1-\boldsymbol{\alpha }){{\varvec{f}}}_{-}({\varvec{x}})$$

where ${\varvec{x}}$ denotes a set of vectors of attribute values, $\alpha =p(y=1)$ is the class prior, *f* is the probability density function of the true distribution, and ${f}_{+}$ of the positive and ${f}_{-}$ of the negative examples [2].

While PU learners also aim to classify positive and negative examples subject to their attributes, they must do so from the labeled subset of positive examples and unlabeled data instances, i.e., they cannot learn from negative examples.

The binary variable $s$ indicates whether $x$ was selected to be labeled [5]. In the PU data setting, the probability of an example to be selected for labeling equals 0 if it is unlabeled in the dataset:

2 $${\varvec{p}}\left({\varvec{s}}=1\right|{\varvec{x}},{\varvec{y}}=0)=0$$

Accordingly, if an example was selected for labeling, it belongs to the positive class:

3 $${\varvec{p}}\left({\varvec{y}}=1\right|{\varvec{s}}=1)=1$$

A PU dataset can then be described as a set of triplets $(x, y, s)$. Note also that only $(x,s)$ are recorded and that if $s=0$, an example could be positive or negative [5, 28].

Bekker et al. [27] define the propensity score, i.e., the probability for a positive example $x$ to be selected for labeling, as $e(x) = p(s=1|x,y=1)$. Building on the PU definition and Bayes’ rule, the authors then define the labeled distribution ${f}_{l}$ in relation to the positive distribution ${f}_{+}$ as

4 $${{\varvec{f}}}_{{\varvec{l}}}\left({\varvec{x}}\right)=\frac{{\varvec{e}}({\varvec{x}})}{{\varvec{c}}}{{\varvec{f}}}_{+}\left({\varvec{x}}\right)$$

with label frequency $c=p(s=1|y=1)$, describing the fraction of positive examples selected for labeling.

#### Assumptions

a. Training set

We assume a single-training-set scenario [2]. This means that both positive and unlabeled examples originate from the same dataset. Additionally, the dataset is an iid sample from the real distribution. Further assuming that a fraction $c$ from the positive instances were selected for labeling according to their individual propensity score $e(x)$, the share of labeled examples in the dataset is $\alpha e(x)$ in the single-training-set scenario.

$${\varvec{x}} \sim f(x)$$

5 $$\sim \boldsymbol{\alpha }{\varvec{e}}({\varvec{x}}){{\varvec{f}}}_{{\varvec{l}}}\left({\varvec{x}}\right)+(1-\boldsymbol{\alpha }{\varvec{e}}({\varvec{x}})){{\varvec{f}}}_{{\varvec{u}}}({\varvec{x}})$$

with ${f}_{l}$ and ${f}_{u}$ denoting the labeled and unlabeled distribution, respectively.

We assume a single-training-set scenario as positive and unlabeled examples of sepsis cases recorded in hospital administrative data all originate from the Hospital Case Cost Statistic dataset provided by the Swiss Federal Statistics Office. Data for this dataset are collected at all hospital sites in Switzerland. Recording of positive or unlabeled examples is not limited to a subset of hospitals but each hospital can record both positive as well as negative (or rather unlabeled) instances. Single-training-set scenarios are most common in PU learning applications and thus, available learning algorithms can usually manage them well [2].

b. Labeling mechanism

To enable learning from PU data, the mechanism how an example was selected to be labeled as positive must be understood for one’s dataset [2, 28]. As outlined earlier, we must assume a bias in diagnosis, documentation, and coding of sepsis cases. First, sepsis might not have been diagnosed as, for instance, physicians did not interpret symptoms accordingly and thus, for instance, did not order laboratory analyses and/ or did not take other diagnostic measures. Whether sepsis was diagnosed or not thus is biased by treating physicians’ experience with sepsis as a clinical condition and their behavior and decisions when observing symptoms. Second, coding relies on thorough documentation. Schwarzkopf et al. [7] found that if sepsis was not named in patients’ medical charts, only 7.6% of true positive cases were explicitly coded as sepsis-1 cases while of the cases where sepsis was named in the medical chart, 61.8% of true positive cases were explicitly coded. Third, the findings of Schwarzkopf et al. [7] also show that even if documented correctly, 38.2% of true positive cases were not coded, implying improvement potential in coding practices. According to Schwarzkopf et al. [7], documentation and coding quality varied strongly between the ten hospitals participating in their study, potentially underscoring how the experience and behavior of doctors, nurses, and coding specialists influence data quality.

Regarding labeling mechanisms, it is either assumed that positive examples were Selected Completely At Random (SCAR) or Selected At Random (SAR) [2]. In our dataset, the attributes potentially biasing diagnosis, documentation, and coding of sepsis cases are not recorded. For instance, there is no attribute describing physicians’ experience with sepsis cases or qualification of coding specialists translating documented information into ICD-10-codes. Still, we must follow the SAR assumption: positive examples’ probability to be labeled in our dataset depends on their attributes, i.e., our sample is a biased sample from the positive distribution [2, 28]:

6 $$e\left(x\right)=p\left(s=1\right|x, y=1)$$

Bekker et al. [27] introduced different settings in which learning under the SAR assumption with unknown exact propensity scores is possible. Specifically, in the context of our study, we may assume a reduction of SAR to SCAR as the attributes influencing the examples’ propensity score to be labeled are fewer (and in fact different) than the attributes used for the classification model. The underlying additional assumption is that it is not possible to know if an example got labeled due to its propensity score or due to a low class probability. Then, there must be a subset of attributes the propensity score depends upon, i.e., the propensity attributes ${x}_{e}$:

$$p\left(s=1\right|x, y=1)=p\left(s=1\right|{x}_{e}, y=1)$$

7 $$e\left(x\right)=e({x}_{e})$$

In the hospital context, attributes influencing the labeling mechanism are the experience and behavior of physicians, nurses, and coding specialists (and the written and verbal communication between these groups). We may assume a reduction from SAR to SCAR, as the classification model will rely on other attributes than the labeler (i.e., the coding specialist). Specifically, hospital cost data are irrelevant for coding of diagnoses (and DRGs) at case level and thus, the labeler does not use these data.

c. Data and class distribution

Lastly, we assume separability and smoothness regarding data distribution:

- Separability: A classifier exists that can distinguish between negative and positive examples. Bekker and Davis [2] formulate this assumption as a function $f$ mapping positive examples to a value above or equal to a defined threshold $\tau$ and vice versa for negative examples:
  8 $$f\left({x}_{i}\right)\ge \tau , {y}_{i}=1$$
  9 $$f\left({x}_{i}\right)<\tau , {y}_{i}=0$$

We expect hospital cost data to be a rich data source for training such a classifier.

- Smoothness: If the attributes of two instances ${x}_{1}$ and ${x}_{2}$ are similar, their probability to belong to the positive class will also be similar, i.e. $p(y=1|{x}_{1}$) is similar to $p(y=1|{x}_{2}$) [2]. As outlined earlier, we assume cost attributes to reflect treatment of sepsis symptoms (e.g., medication, ventilation, intensive care nursing, etc.). Thus, cases with similar cost patterns should have a similar probability to belong to the positive sepsis class.

In the results section, we present a cumulative distribution function supporting both the separability and the smoothness assumption.

### Applied learning techniques

PU learning algorithms can be categorized into (1) one-class, (2) heuristic, (3) robust, and (4) bias-based approaches. Each of these approaches has distinct advantages and disadvantages, well-described elsewhere [2, 29, 30]. PU learning originates from text and document classification and bioinformatics research. In this study, we employ one PU learning approach from each field to investigate their suitability for and transferability to a different field, namely hospital administrative data.

The first PU learning approach that we employ, the “spy” approach, was originally developed by Liu et al. [31] for text and document classification. This approach belongs to the family of heuristic, two-step approaches. In a first step, the aim of such approaches is to learn reliable negatives examples via a heuristic. To this end, for the spy approach, positive examples are added to the unlabeled data. These serve as “spies” in the unlabeled data. We use 10% of our positive examples as spies. Subsequently, a classifier is employed treating positive examples as positives and unlabeled instances, including the spies, as negatives. The lowest prediction probability of any spy is then used as a threshold to identify reliable negative examples. Concretely, all unlabeled instances with a lower prediction probability than the lowest prediction probability of any spy will be labeled as negative. For the second step, the spies are added back to the positive examples and both positive and negative examples are used to classify the remaining unlabeled instances in a supervised learning environment.

In their study, Liu et al. [31] do not use the lowest prediction probability of any spy as the threshold for identifying reliable negative examples due to noise in their data. Instead, they derive the threshold by defining four different shares of examples (5%, 10%, 15%, and 20%) that should be reliable negatives according to the threshold. We opt for a different strategy: We run the first step 1,000 times for each cross-validation fold (cf. Model evaluation below). We then average the minimum prediction probabilities of the 1,000 runs.

We employ the spy approach with the default classifiers of the two step spy naïve bays Python notebook by Kiyomaru et al. [32], i.e., logistic regression for the first and naïve Bayes for the second step. Naïve bays is a straightforward classifier and serves as our baseline model. In addition, we update the spy approach with a state-of-the-art classifier for both steps, specifically gradient boosted trees, employing the XGBoost package [33], version 2.1.4.^1^ For the spy approach, we performed all calculations with the Python version 3.12.0.

As second PU learning approach, we rely on the AdaSampling package for the programming language *R*, developed by Yang et al. [28, 29]. AdaSampling was developed building on wrapper-based feature selection [34]. It belongs to the PU learning algorithm category of robust approaches, yet it does not identify negative instances via noisy filtering or a prespecified threshold, nor does it need bias estimation like other robust approaches.

Instead, Yang et al. [28] outline that the algorithm iteratively estimates the probability of data instances’ labels to be mislabeled using a selectable learning algorithm, such as radial kernel support vector machine (SVM) or k-nearest-neighbors (k-NN). With each new iteration, the estimation is based on a resampled dataset from the initial sample with the probability of a data instance to be excluded from this resampled dataset being equal to its probability to be mislabeled. This means that data instances with a higher probability to be mislabeled are more likely to be eventually excluded from updated training sets. The training set for the final prediction of the binary classifier then consists of examples with a relatively low probability to be mislabeled, i.e., they are reliable examples.

The AdaSampling algorithm has demonstrated performance equivalent to or better than that of other methods when applied to real-world data, e.g., in terms of sensitivity [35] or when estimating positive prediction probability for unlabeled positive examples [36], yet Zhou et al. [37] found AdaSampling to be inferior to their problem-specific algorithm in terms of Area Under the ROC curve when applied to synthetic data.

We leverage dozens of hospital cost categories as our main learning attributes. As outlined above, we hypothesize that treatment of both labeled and unlabeled sepsis cases will incur similar costs in terms of categories (e.g., for physicians working in the intensive care unit) as well as cost level per category. In other words, cost patterns of labeled and unlabeled sepsis cases should be similar. We hypothesize that classification algorithms that perform well for pattern recognition should therefore be able to utilize hospital cost data well for our learning task.

SVM has several other important strengths[38], which are relevant to our setting. These include good performance in multi-dimensional feature space and strong generalization ability, and especially for radial kernel SVM no need for parametric assumptions, learning of non-linear decision boundaries, and recognition of complex feature interaction. Lastly, although SVM does not provide explicit feature importance scores, it implicitly emphasizes informative features by shaping decision boundaries based on relevant patterns in the data.

Therefore, from the set of classification algorithms available for AdaSampling – SVM, k-NN, feature weighted k-NN, logistic regression, and linear discriminant analysis – we chose SVM for our classification task. We operationalize the SVM as a radial kernel SVM to optimize detection of non-linear relationships. For many hospital cost categories, variation is large. Therefore, we normalize feature values (min–max normalization) to enhance learning results for the radial kernel SVM [39]. We run the SVM classifier 20 times, inducing an ensemble learning model.

For AdaSampling, we performed all calculations with the *R* version 4.2.1 2021.11.01 and the AdaSampling version 1.3.

### Model evaluation

To answer our research question, we need to assess the quality of the PU learners’ output. The main challenge is that by PU definition, real-world datasets do not contain actual negatives. Thus, false positives (FP) and true negatives (TN) cannot be identified in PU data. Commonly, when developing PU learners, authors circumvent this evaluation issue creating synthetic datasets and/ or using benchmark datasets, e.g., when evaluating developed algorithms.

Our goal is to increase the quality of a real-world dataset, however. Hence, we must use the actual dataset in which only actual positive examples are reliable. Combined with the positive and negative predictions from our model, we can calculate recall (equal to sensitivity) $r$, recall at k% ${r}_{k}$ and precision at k% ${p}_{k}$ [40, 41] for evaluation:

10 $$r=\frac{TP}{TP+FN}$$

11 $${r}_{k}=\frac{TP\;in\;top\;k{\%}}{all\;TP\;in\;dataset}$$

12 $$p_k=\frac{TP\;in\;top\;k\%}{all\;data\;instances\;in\;top\;k\%}$$

As Bekker et al. [2] point out, recall can be estimated in the PU learning setting under the SCAR assumption, which is why it is essential that we can reduce SAR to SCAR as discussed earlier. We argue that for our goal, recall is the central evaluation metric as we need to correctly identify as many positive sepsis cases from the administrative data as possible.

We estimate ${r}_{k}$ and ${p}_{k}$ for the top 10%, 20%, and 30% of predictions. Both metrics were developed for information retrieval applications such as web searches [40, 41]. With ${r}_{k}$, we can evaluate what share of TPs in the total dataset are among the top k% of our models’ predictions. With ${p}_{k}$, we assess the share of TPs in the top k% of our models’ predictions.

For evaluation, we perform cross-validation with five equal folds. We use the positive class label means of each cross validation fold as threshold for positive classification. When evaluating, we calculate the above metrics counting actual positive examples in five different constellations: (1) All positive cases according to the explicit coding strategy (main model), (2) cases that are positive according to both the explicit and implicit coding strategy, (3) all positive cases according to the implicit coding strategy, (4) cases positive according to the explicit or the implicit coding strategy, and (5) cases positive only according to the implicit coding strategy (see Fig. 3 in the supplements). Note that we train our model defining positive labels according to the explicit coding strategy. Constellations (2) to (5) will thus help us to evaluate whether our learner can correctly identify positive examples of alternative sepsis coding strategies in the unlabeled data.

### External validity check

While using the positive class label mean as classification threshold is standard in machine learning, it might not be adequate in the application context of learning positive sepsis cases from PU hospital administrative data. When applying our learned models, unlabeled sepsis cases with high model prediction probabilities would be relabeled as positive. As a result, a new sepsis rate emerges. Whether this new sepsis rate is more or less realistic than the initial, raw sepsis rate depends on the classification threshold. Moreover, it conveys information about the false positive predictions of our model, as relatively high sepsis rates would indicate that our model classifies unrealistically many unlabeled cases as positive. Thus, we check the external validity of our results as central part of our model evaluation.

To this end, we derive new sepsis rates relabeling unlabeled examples as positive sepsis cases and compare them to sepsis rates estimated from medical record review studies. For relabeling, we define classification thresholds for relabeling unlabeled examples based on the top 5%, 10%, 15%, 20%, 25%, and 30% of prediction values. The positive class label mean classification threshold of our main model serves as comparison. Subsequently, we estimate new sepsis rates ${s}_{k}$ and ${s}_{\widehat{y}=1}$ after relabeling, with $k$ indicating the top k% of predictions and with $\widehat{y}=1$ indicating all positive predictions according to the positive class label mean.

Due to undersampling, we need to transfer the rate of relabeled sepsis cases in the unlabeled examples in our final sample of 313,434 cases to learn the PU classifier to the initial sample of more than 2.6 million cases:

13 $${s}_{k}=\frac{{l}_{i}+{u}_{i}\times {r}_{k}/{u}_{t}}{{l}_{i}+{u}_{i}}$$

14 $${s}_{k}=\frac{{s}_{k,i}}{{n}_{i}}$$

and ${s}_{\widehat{y}=1}$ formulated likewise, with ${l}_{i}$ expressing the number of labeled positive cases (explicit coding) in the initial sample $i$, ${u}_{i}$ the number of unlabeled examples in $i$, ${r}_{k}$ the number of relabeled unlabeled instances according to $k$ (and ${r}_{p}$ the number of relabeled unlabeled instances according to the class label mean), and ${u}_{t}$ the number of unlabeled examples in our final sample $t$. With equation (13), we apply the rate of relabeled sepsis cases in the unlabeled data in our final sample ${r}_{k}/{u}_{t}$ to all unlabeled instances in the initial sample ${u}_{i}$ and add labeled positive sepsis cases ${l}_{i}$. This yields equation (14), i.e., the total number of sepsis cases ${s}_{k,i}$ divided by all cases ${n}_{i}$ to receive the new sepsis rate ${s}_{k}$ or ${s}_{\widehat{y}=1}$.

Studies indicate that the actual sepsis rate after medical record review is 2.4 to 4.1 times higher than raw rates estimated from hospital administrative data [7, 8]. As external validity check, we compare this factor range with the ratio of ${s}_{k}$ or ${s}_{\widehat{y}=1}$ and our initial, raw sepsis rate of 2.40%.

## Results

### Descriptive results

We present descriptive results of selected attributes in Table 1 and of the number and share per ICD-10-chapter in Table 2. We provide a full list of descriptive results in Table 7 in the supplements.

**Table 1:** Descriptive results of selected attributes

Attribute	Total sample (n = 313,433)		Labeled sepsis cases (n = 63,434)		Unlabeled cases (n = 250,000)	
	Mean (SD)	Median (25th – 75th)	Mean (SD)	Median (25th – 75th)	Mean (SD)	Median (25th – 75th)
Total costs	17,260 (37,739)	8,537 (4,953–16,319)	37,098 (72,376)	15,186 (8,013–35,426)	12,226 (18,201)	7,626 (4,464–13,665)
*Variable direct costs [CHF] – selected attributes*						
Pharmaceuticals	479 (2,938)	87 (20–242)	1,428 (5,846)	271 (100–841)	238 (1,366)	67 (12–173)
Blood and blood products	204 (2,846)	0 (0–0)	779 (6,003)	0 (0–0)	58 (953)	0 (0–0)
Medical material	576 (1,974)	81 (1–428)	1,029 (3,484)	113 (18–615)	461 (1,318)	73 (0–397)
*Fixed indirect costs [CHF] – selected attributes*						
Operating room, overheads excl. IUC	714 (2,106)	0 (0–886)	1,003 (3,806)	0 (0–34)	641 (1,364)	0 (0–964)
Operating room, IUC	183 (655)	0 (0–212)	238 (842)	0 (0–2)	170 (597)	0 (0–235)
Operating room doctors—activities 6a, overheads excl. IUC	237 (873)	0 (0–8)	349 (1,402)	0 (0–0)	209 (673)	0 (0–57)
Operating room doctors—activities 6a, IUC	13 (68)	0 (0–0)	19 (108)	0 (0–0)	11 (54)	0 (0–0)
Anesthesia, overheads excl. IUC	601 (1,565)	0 (0–790)	867 (2,788)	0 (0–514)	533 (1,037)	0 (0–825)
Anesthesia, IUC	58 (152)	0 (0–72)	81 (266)	0 (0–42)	52 (104)	0 (0–76)
Intensive care unit, overheads excl. IUC	1,919 (13,374)	0 (0–0)	7,763 (27,900)	0 (0–3,495)	437 (3,986)	0 (0–0)
Intensive care unit, IUC	181 (1,251)	0 (0–0)	730 (2,601)	0 (0–293)	42 (386)	0 (0–0)
Intensive care unit physicians—activities 6b1, overheads excl. IUC	365 (3,003)	0 (0–0)	1,448 (6,294)	0 (0–181)	90 (940)	0 (0–0)
Intensive care unit physicians—activities 6b1, IUC	15 (149)	0 (0–0)	57 (314)	0 (0–0)	4 (49)	0 (0–0)
IMCU, overheads excl. IUC	269 (2,844)	0 (0–0)	818 (5,735)	0 (0–0)	129 (1,304)	0 (0–0)
Intermediate Care Units (IMCU), IUC	19 (231)	0 (0–0)	57 (461)	0 (0–0)	10 (112)	0 (0–0)
IMCU physicians—activities 6b2, overheads excl. IUC	38 (431)	0 (0–0)	110 (812)	0 (0–0)	20 (254)	0 (0–0)
IMCU physicians—activities 6b2, IUC	2 (34)	0 (0–0)	5 (46)	0 (0–0)	1 (31)	0 (0–0)
Emergency, overheads excl. IUC	185 (323)	0 (0–307)	351 (370)	306 (85–478)	143 (296)	0 (0–231)
Emergency, IUC	19 (36)	0 (0–30)	36 (41)	26 (6–53)	15 (34)	0 (0–21)
Emergency medical services—activities 6b3, overheads excl. IUC	89 (186)	0 (0–117)	166 (227)	67 (0–279)	69 (168)	0 (0–41)
Emergency medical services—activities 6b3, IUC	4 (12)	0 (0–1)	7 (15)	0 (0–7)	3 (10)	0 (0–0)
Imaging procedures, overheads excl. IUC	277 (697)	41 (0–291)	668 (1190)	305 (87–758)	178 (448)	0 (0–179)
Imaging procedures, IUC	77 (201)	8 (0–76)	182 (331)	77 (24–203)	50 (140)	0 (0–47)
Laboratory, overheads excl. IUC	476 (1,755)	100 (0–371)	1,505 (3,494)	584 (260–1,303)	215 (652)	60 (0–214)
Laboratory, IUC	56 (217)	10 (0–40)	178 (432)	62 (24–151)	25 (84)	6 (0–23)
Dialysis, overheads excl. IUC	60 (1020)	0 (0–0)	244 (2161)	0 (0–0)	13 (330)	0 (0–0)
Dialysis, IUC	7 (115)	0 (0–0)	26 (241)	0 (0–0)	2 (42)	0 (0–0)
Physicians, activities 1–5, overheads excl. IUC	1,378 (3,084)	783 (223–1,579)	2,614 (5,393)	1,481 (785–2,779)	1,065 (2,014)	647 (131–1,315)
Physicians, activities 1–5, IUC	95 (229)	37 (2–103)	173 (363)	78 (26–179)	75 (175)	29 (0–87)
Physiotherapy, overheads excl. IUC	255 (1,096)	0 (0–200)	623 (1,845)	196 (0–615)	161 (774)	0 (0–124)
Physiotherapy, IUC	34 (202)	0 (0–24)	74 (192)	21 (0–76)	23 (203)	0 (0–14)
Nursing care, overheads excl. IUC	3,606 (7,742)	1,577 (760–3,590)	7,322 (12,556)	3,801 (1,741–8,210)	2,663 (5,546)	1,328 (681–2,717)
Nursing care, IUC	251 (604)	104 (39–247)	473 (954)	217 (87–511)	194 (459)	88 (35–199)
*CHOP chapters: Procedures and surgeries of the…*						
Nervous system	0.09 (0.45)	0 (0–0)	0.08 (0.59)	0 (0–0)	0.09 (0.41)	0 (0–0)
Endocrine system	0 (0.07)	0 (0–0)	0 (0.05)	0 (0–0)	0 (0.08)	0 (0–0)
Eye	0.02 (0.25)	0 (0–0)	0 (0.07)	0 (0–0)	0.02 (0.27)	0 (0–0)
Ears	0.01 (0.11)	0 (0–0)	0 (0.07)	0 (0–0)	0.01 (0.11)	0 (0–0)
Nose, mouth, throat	0.04 (0.32)	0 (0–0)	0.02 (0.24)	0 (0–0)	0.04 (0.33)	0 (0–0)
Respiratory system	0.07 (0.52)	0 (0–0)	0.23 (0.96)	0 (0–0)	0.04 (0.31)	0 (0–0)
Cardiovascular system	0.29 (1.55)	0 (0–0)	0.68 (2.85)	0 (0–0)	0.19 (0.95)	0 (0–0)
Hematopoietic and lymphatic system	0.02 (0.19)	0 (0–0)	0.04 (0.26)	0 (0–0)	0.02 (0.17)	0 (0–0)
Digestive tract	0.28 (1.19)	0 (0–0)	0.68 (2.24)	0 (0–0)	0.18 (0.67)	0 (0–0)
Urinary organs	0.1 (0.46)	0 (0–0)	0.22 (0.68)	0 (0–0)	0.07 (0.37)	0 (0–0)
Male sexual organs	0.01 (0.15)	0 (0–0)	0.01 (0.16)	0 (0–0)	0.02 (0.14)	0 (0–0)
Female sexual organs	0.05 (0.33)	0 (0–0)	0.01 (0.16)	0 (0–0)	0.05 (0.36)	0 (0–0)
Obstetric procedures	0.11 (0.5)	0 (0–0)	0 (0.08)	0 (0–0)	0.14 (0.56)	0 (0–0)
Musculoskeletal system	0.29 (1.05)	0 (0–0)	0.22 (1.24)	0 (0–0)	0.31 (0.99)	0 (0–0)
Integumentary system	0.14 (1.14)	0 (0–0)	0.38 (2.2)	0 (0–0)	0.08 (0.62)	0 (0–0)
Other diagnostic or therapeutic procedures	2.44 (5.67)	1 (0–2)	3.21 (4.78)	2 (0–4)	2.24 (5.86)	0 (0–1)
Measurement instruments	0.84 (5.97)	0 (0–0)	0.66 (5.44)	0 (0–0)	0.88 (6.1)	0 (0–0)
Rehabilitation	0.02 (0.14)	0 (0–0)	0.01 (0.11)	0 (0–0)	0.02 (0.15)	0 (0–0)
Procedures not classified elsewhere	0.25 (0.77)	0 (0–0)	0.31 (0.95)	0 (0–0)	0.23 (0.71)	0 (0–0)
*Sample descriptives not used for classification (Age, gender, Elixhauser Comorbidity Index Score), and additional descriptive information (mortality, positive class label mean)*						
Age	55.1 (27.1)	60 (35–75)	69.3 (20.3)	75 (60–85)	51.5 (27.4)	55 (30–75)
Share of female patients	49.19%		57.52%		47.07%	
Elixhauser Comorbidity Index Score	4.9		11.8		3.2	
Number of unweighted Elixhauser diagnoses	2.0		3.9		1.5	
Inpatient mortality rate	3.89%		13.99%		1.33%	
Positive class label mean	0.2024					

Annotations: CHOP = Swiss Operation and Procedure Catalogue; IMCU = Intermediate Care Unit; IUC = Infrastructure Usage Costs. All costs are in Swiss francs and rounded to full numbers. All shares, percentages, and CHOP chapters are rounded to two decimals. Age is rounded to one decimal. Labeled sepsis cases follow the explicit coding definition presented in Table 5 in the supplements. We calculated the Elixhauser Comorbidity Index Score according to Van Walraven et al. [42]. The inpatient mortality rate is not risk-adjusted

Source: Hospital Case Cost Dataset from the Swiss Federal Statistics Office, data years 2017 to 2019

**Table 2:** Descriptive results of ICD chapters of cases’ main diagnosis

ICD chapter	ICD chapter name	Total sample (n = 313,433)		Labeled sepsis cases (n = 63,434)		Unlabeled cases (n = 250,000)	
		Number	Share	Number	Share	Number	Share
A00-B99	Certain infectious and parasitic diseases	29,728	9.5%	24,924	39.3%	4,804	1.9%
C00-D49	Neoplasms	26,067	8.3%	5,162	8.1%	20,905	8.4%
D50-D90	Diseases of the blood and blood-forming organs and certain disorders involving the immune mechanism	1,400	0.4%	222	0.3%	1,178	0.5%
E00-E89	Endocrine, nutritional, and metabolic diseases	4,945	1.6%	583	0.9%	4,362	1.7%
F00-F99	Mental and behavioral disorders	19,800	6.3%	431	0.7%	19,369	7.7%
G00-G99	Diseases of the nervous system	7,203	2.3%	608	1.0%	6,595	2.6%
H00-H59	Diseases of the eye and adnexa	1,607	0.5%	12	0.0%	1,595	0.6%
H60-H95	Diseases of the ear and mastoid process	1,470	0.5%	26	0.0%	1,444	0.6%
I00-I99	Diseases of the circulatory system	29,330	9.4%	3,512	5.5%	25,818	10.3%
J00-J99	Diseases of the respiratory system	23,642	7.5%	7,745	12.2%	15,897	6.4%
K00-K93	Diseases of the digestive system	26,412	8.4%	4,771	7.5%	21,641	8.7%
L00-L99	Diseases of the skin and subcutaneous tissue	3,726	1.2%	610	1.0%	3,116	1.2%
M00-M99	Diseases of the musculoskeletal system and connective tissue	29,089	9.3%	1,268	2.0%	27,821	11.1%
N00-N99	Diseases of the genitourinary system	22,379	7.1%	8,566	13.5%	13,813	5.5%
O00-O99	Pregnancy, childbirth, and the puerperium	20,593	6.6%	289	0.5%	20,304	8.1%
P00-P96	Certain conditions originating in the perinatal period	7,786	2.5%	868	1.4%	6,918	2.8%
Q00-Q99	Congenital malformations, deformations, and chromosomal abnormalities	2,197	0.7%	149	0.2%	2,048	0.8%
R00-R99	Symptoms, signs, and abnormal clinical and laboratory findings, not elsewhere classified	7,893	2.5%	532	0.8%	7,361	2.9%
S00-T98	Injury, poisoning and certain other consequences of external causes	34,381	11.0%	3,145	5.0%	31,236	12.5%
U50-U52	Functional impairment	1	0.0%	0	0.0%	1	0.0%
Z00-Z99	Factors influencing health status and contact with health services	13,785	4.4%	11	0.0%	13,774	5.5%

Annotations: ICD = International Classification of Diseases. Shares are rounded to one decimal. Labeled sepsis cases follow the explicit coding definition presented in Table 5 in the supplements. As only one case had a main diagnosis of the ICD chapter U50-U52, we dropped this variable to enable the SVM classifier

Source: Hospital Case Cost Dataset from the Swiss Federal Statistics Office, data years 2017 to 2019

Labeled sepsis cases exhibit more than three times higher total costs than unlabeled cases. Mean costs associated with surgery (operating room cost attributes), the intensive care and intermediate care units, the emergency unit, medical and diagnostic services, and nursing care are all considerably higher for labeled sepsis cases compared to unlabeled cases. Patients labeled as sepsis cases are on average roughly 18 years older than unlabeled cases. The raw inpatient mortality rate is more than ten times higher, and the Elixhauser Comorbidity Index Score is almost four times greater for labeled than for unlabeled cases. Labeled sepsis cases on average receive considerably more surgeries of the respiratory and cardiovascular systems, and of the digestive tract. This is only partially mirrored in Table 2 showing a higher share of main diagnoses in ICD chapter J (diseases of the respiratory system) for labeled sepsis cases (12.2%) than for unlabeled cases (6.4%).

### Model evaluation

We present the results of the model evaluations in Table 3. We train our models with positive examples according to the explicit coding strategy (see Table 5 in the supplements for coding strategies). In the second column of Table 3, positive examples in the test set are labeled according to this explicit coding strategy and compared with model predictions for model evaluation. To receive additional insights into the performance of our model, we follow four alternative strategies for coding sepsis cases as positive examples yielding four additional evaluation scenarios (see Methods section above and Fig. 3 in the supplements). In the second column, we label examples as positive if they are positive according to both the explicit and implicit coding strategy. In the third column, we label an example as positive according to the implicit coding strategy. These examples might also be positive according to the explicit coding strategy but do not have to be. In the fourth column, positive labels are given to cases in the test set that are positive according to the explicit or the implicit coding strategy. In the fifth column, examples are labeled as positive examples exclusively according to the implicit coding strategy.

**Table 3:** Evaluation metrics

Evaluation metric	Explicit	Explicit and implicit	Implicit	Explicit or implicit	Only implicit
*Model learned with “spy” approach, logistic regression for first and naïve bayes classifier for second step*					
Recall	0.933	0.932	0.940	0.939	**0.970**
Recall at 10%	0.117	0.117	0.117	0.117	**0.118**
Recall at 20%	0.236	0.235	0.235	0.236	**0.237**
Recall at 30%	0.355	0.356	0.356	0.355	**0.356**
Precision at 10%	0.220	0.144	0.188	**0.265**	0.045
Precision at 20%	0.223	0.144	0.189	**0.267**	0.045
Precision at 30%	0.224	0.146	0.191	**0.269**	0.045
*Model learned with “spy” approach, XGBoost for both steps*					
Recall	0.998	**1.000**	0.998	0.997	0.992
Recall at 10%	0.282	**0.297**	0.284	0.275	0.238
Recall at 20%	0.564	**0.594**	0.567	0.550	0.474
Recall at 30%	0.814	**0.849**	0.815	0.796	0.700
Precision at 10%	0.569	0.392	0.484	**0.662**	0.092
Precision at 20%	0.570	0.392	0.484	**0.661**	0.092
Precision at 30%	0.548	0.374	0.464	**0.638**	0.090
*Model learned with AdaSampling*					
Recall	0.886	**0.933**	0.869	0.849	0.654
Recall at 10%	0.461	**0.548**	0.496	0.434	0.246
Recall at 20%	0.807	**0.839**	0.800	0.783	0.614
Recall at 30%	**1.000**	**1.000**	**1.000**	**1.000**	**1.000**
Precision at 10%	0.827	0.675	0.738	**0.890**	0.063
Precision at 20%	0.724	0.517	0.595	**0.802**	0.078
Precision at 30%	0.598	0.411	0.496	**0.683**	0.085
Total number of positive examples	63,434	41,396	53,637	75,675	12,241
Evaluation scenario	A	B	C	D	E

Annotations: Coding strategies are presented in Table 5 in the supplements. Values are rounded to three decimals. Highest values per evaluation metric and learning approach are in bold letters. Positive class label means in the training sets of the five cross-validation folds relevant for classification ranged between 0.2021 and 0.2027 for AdaSampling and between 0.5670 and 0.6136 for spy. Note that the positive class label means for spy are higher, but the train set size smaller as for AdaSampling. This is due to the fact that actual positive examples and reliable negative examples are used for training in a supervised learning environment in the second step of the spy approach, as compared to learning from positive examples and unlabeled instances directly. See Table 8 and Table 9 in the supplements for details

Recall is above 0.84 for all approaches and evaluation scenarios, with the exception of evaluation scenario E for AdaSampling. Thus, it seems that all models are generally good at identifying positive cases in unseen data.

However, especially for the spy approach, the very high recalls suggest that the model might classify too many examples as positives. This is supported by the results for precision at k% for the two models using the spy approach. Both models identify less true positive cases among their top k% of predictions than the model learned with AdaSampling. With respect to recall at k%, the model learned with AdaSampling also outperforms both models learned with the spy approach: Among its top k% of predictions, there is a considerably higher share of true positives, and all true positives are among its top 30% of predictions for any evaluation scenario. Lastly, note that with respect to recall and recall at k%, both models learned with the spy approach seem to perform well also for evaluation scenario E, i.e., where actual positives were labeled exclusively according to the implicit coding strategy for evaluation. Precision at k% is low, however. This might also indicate that too many examples are labeled as positives when employing the spy approach.

**Fig. 1:**
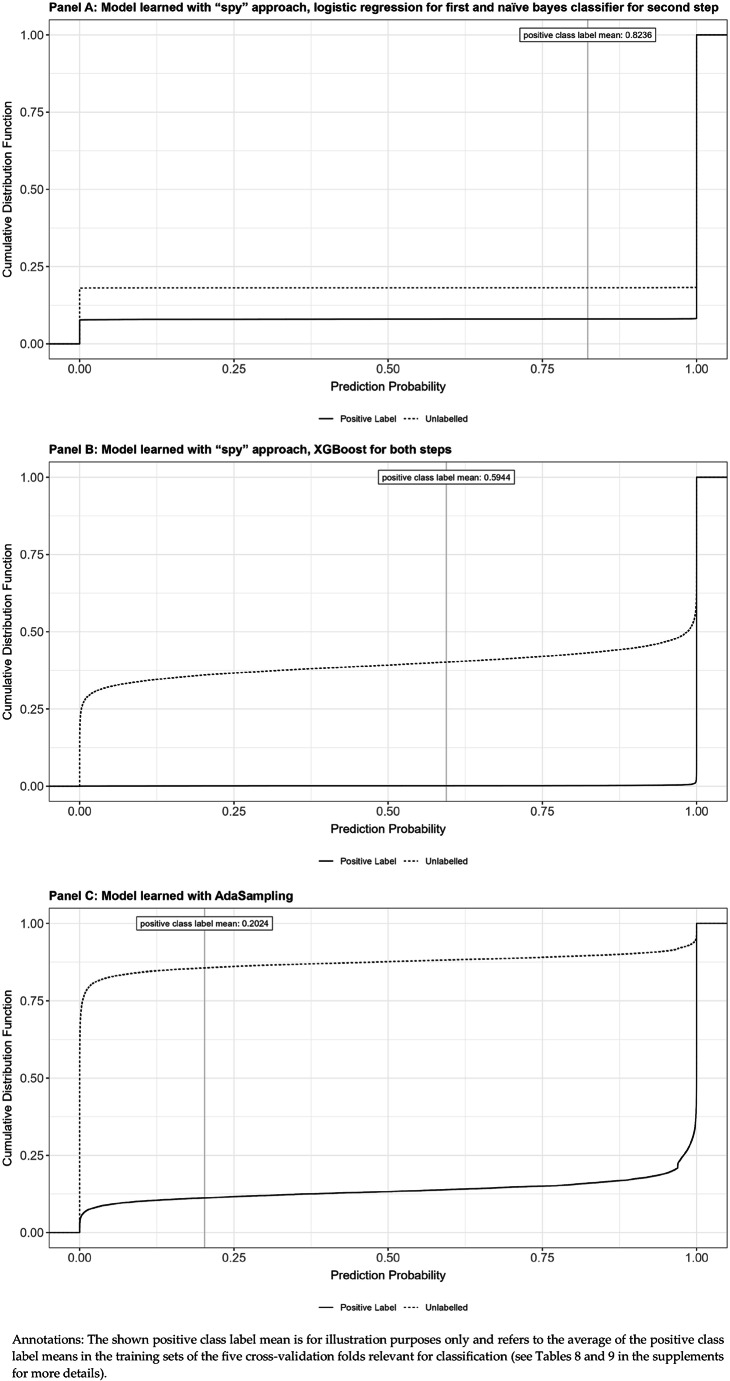
Cumulative distribution function of model predictions by prediction probability

We present cumulative distribution functions of model predictions by prediction probability in Fig. 1.

Consulting Panel A of Fig. 1, we observe that our baseline model learned with the spy approach and a naïve bayes classifier for the second step estimates a prediction probably of 1.0 or close to 1.0 for roughly 93% of positive examples and for roughly 80% of unlabeled data instances. Almost all of the remaining positive examples and unlabeled data instances exhibit a prediction probability of 0 or close to 0. There are hardly any examples with prediction probabilities between these two extremes. The recall of evaluation scenario A of 0.933 is perceptible at the intersect of the line of the positive class label mean and the cumulative distribution function of the positive examples. Panel B shows that our model learned with the spy approach and XGBoost estimates a prediction probably of 1.0 or close to 1.0 for almost all positive examples, and also for roughly 50% of unlabeled data instances. As the evaluation metrics presented above, these results also suggest that too many examples are labeled as positives when employing the spy approach.

Panel C shows that the model learned with AdaSampling estimates a prediction probability of 0 or close to 0 for roughly 80% of unlabeled data instances and for roughly 5% of positive examples. Inversely, our model estimates a prediction probability of 1.0 or close to 1.0 for roughly 5% of unlabeled data instances and for roughly 80% of positive examples. For both unlabeled data instances and positive examples, our model estimates a prediction probability between close to 0 to approximately 0.95 for the remaining approximately 15% of data instances. This indicates that the model learned with AdaSampling seems to respect the model assumptions regarding separability and smoothness best. Lastly, looking at the intersect of the positive class label mean and the cumulative distribution function of the positive examples, the recall of 0.886 of evaluation scenario A becomes evident.

### External validity check

Table 4 presents the results of our external validity check deriving new sepsis rates after relabeling unlabeled examples as positive cases according to different classification thresholds.

**Table 4:** New sepsis rates according to different classification thresholds

Threshold based on…	Classifi-cation threshold	Relabeled examples,$${{\varvec{u}}}_{{\varvec{i}}}\times {{\varvec{r}}}_{{\varvec{k}}}/{{\varvec{u}}}_{{\varvec{t}}}$$	New positive sepsis cases,$${{\varvec{l}}}_{{\varvec{i}}}+{{\varvec{u}}}_{{\varvec{i}}}$$$$\times {{\varvec{r}}}_{{\varvec{k}}}/{{\varvec{u}}}_{{\varvec{t}}}$$	New sepsis rate, $${{\varvec{s}}}_{{\varvec{k}}}$$ or $${{\varvec{S}}}_{\widehat{{\varvec{y}}}=1}$$	Ratio	Recall,$${\varvec{r}}$$	False negatives
*Model learned with “spy” approach, logistic regression for first and naïve bayes classifier for second step*							
Top 30%	1.00000	2,117,175	2,180,609	0.82657	34.4	0.854	9,251
Top 25%	1.00000	2,117,175	2,180,609	0.82657	34.4	0.854	9,251
Top 20%	1.00000	2,117,175	2,180,609	0.82657	34.4	0.854	9,251
Top 15%	1.00000	2,117,175	2,180,609	0.82657	34.4	0.854	9,251
Top 10%	1.00000	2,117,175	2,180,609	0.82657	34.4	0.854	9,251
Top 5%	1.00000	2,117,175	2,180,609	0.82657	34.4	0.854	9,251
Pos. class label mean	0.82357	2,119,667	2,183,101	0.82752	34.4	0.867	8,413
*Model learned with “spy” approach, XGBoost for both steps*							
Top 30%	0.99999	537,721	601,155	0.22787	9.5	0.875	7,902
Top 25%	1.00000	385,916	449,350	0.17033	7.1	0.776	14,202
Top 20%	1.00000	385,916	449,350	0.17033	7.1	0.776	14,202
Top 15%	1.00000	385,916	449,350	0.17033	7.1	0.776	14,202
Top 10%	1.00000	385,916	449,350	0.17033	7.1	0.776	14,202
Top 5%	1.00000	385,916	449,350	0.17033	7.1	0.776	14,202
Pos. class label mean	0.59438	1,539,402	1,602,836	0.60756	25.3	0.998	137
*Model learned with AdaSampling*							
Top 30%	0.14822	390,376	453,810	17.20%	7.2	0.891	6,904
Top 25%	0.87035	262,331	325,765	12.35%	5.1	0.831	10,730
Top 20%	0.99034	177,901	241,335	9.15%	3.8	0.714	18,158
Top 15%	0.99967	110,362	173,796	6.59%	2.7	0.573	27,073
Top 10%	0.99999	63,904	127,338	4.83%	2.0	0.436	35,764
Top 5%	1.00000	50,176	113,610	4.31%	1.8	0.389	38,760
Pos. class label mean	0.20238	373,960	437,394	16.58%	6.9	0.886	7,233

Annotations: Pos. = Positive. The classification threshold is based on the top-k% of model predictions or the positive class label mean, and it represents the smallest prediction probability an unlabeled example needed to have to be relabeled (average across the five cross validation folds is shown). The ratio presents the ratio of ${s}_{\text{k}}$ or ${{\varvec{S}}}_{\widehat{{\varvec{y}}}=1}$ with the initial, raw sepsis rate in the initial sample of 2.40%. Recall is calculated according to the classification threshold and evaluation scenario A. The column false negatives shows the number of false negative predictions when using the indicated classification threshold. All values are rounded to the shown number of decimals

Regarding the spy approach, the classification threshold is 1.0 or very close to 1.0 for all top-k% prediction probabilities. The results for the model learned with the spy approach and logistic regression for the first and naïve bayes classifier for the second step show clearly that while recall is above 0.854 for all constellations, the number of relabeled cases and emerging new sepsis rates are very high and unrealistic. This finding is in line with the cumulative distribution function of prediction probabilities (cf. Panel A of Fig. 1 above). For most constellations, the model learned with the spy approach and XGBoost for both steps has a recall of 0.776 and relabels roughly 386,000 cases, yielding a new sepsis rate of 17.03%, which is more than 7 times higher than the initial sepsis rate of 2.40%.

With regards to the model learned with AdaSampling, the classification threshold is greater than 0.99 when defined by the top 20% or higher of prediction probabilities. The classification threshold based on the positive class label mean is in between the threshold based on the top 25% and top 30% of model predictions. More unlabeled examples are relabeled with a decreasing classification threshold. The same dynamic is true for the new sepsis rate. Recall is highest when basing the classification threshold on the top 30% of model predictions (0.891) and lowest for the top 5% classification threshold (0.389). The ratio between the new sepsis rate and the initial, raw sepsis rate is between 1.8 and 7.2.

**Fig. 2:**
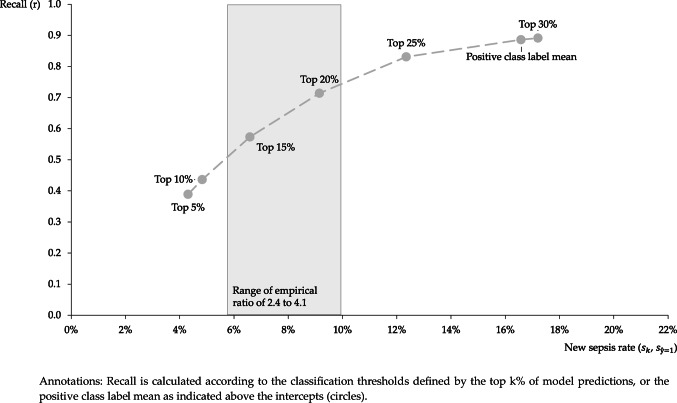
Relationship between the new sepsis rates and recalls

Figure 2 presents the relationship between the new sepsis rates and recalls supplied by the model learned with AdaSampling. We limit this visualization to the results from the model learned with AdaSampling, as new sepsis rates resulting from the external validity checks of the other models are substantially higher as potential new sepsis rates derived from medical record review studies, and new sepsis rates and recalls do not vary (enough) between different top-k% prediction probability thresholds.

## Summary and discussion

### Contributions

To the best of our knowledge, we are the first to assess whether PU learning is an efficient approach to increase the quality of hospital administrative data. We rely on sepsis as application example as it is commonly underreported in hospital administrative data. We focus on hospital cost data for learning our models as even if undiagnosed, not documented, and/ or not coded, sepsis symptoms will always have to be treated, producing specific treatment cost patterns. Our model evaluation and external validity checks imply that PU learning can indeed serve as an approach to identify and relabel positive sepsis cases part of the unlabeled data, thereby increasing the quality of hospital administrative data. However, our findings underline that the quality and potential for further downstream use of model outputs depend on the chosen PU learning approach and classification algorithm.

Our contributions to the PU learning literature are that we updated the spy approach with a state of the art classifier, and that we developed an external validity check capable of providing insights to assess model outputs’ quality, addressing the challenge of limited evaluation possibilities in PU learning applications with real-world data. Furthermore, our study shows that some out-of-the-box methods, specifically AdaSampling, originally developed for a different research field, are potentially able to effectively classify unlabeled sepsis cases. This could have implications for quantitative studies in the fields of health services research, health economics, and healthcare management science, and for disease surveillance, employing hospital administrative data. Our findings suggest that potentially, in such studies, PU learners could be employed to relabel unlabeled sepsis cases for downstream (sensitivity) analyses.

### Summary of experimental results

When exclusively considering recall, our evaluation results suggest that all models perform equally well or even that the two-step spy approach works better than the robust approach. Still, the very high recalls of the two models learned with the spy approach for evaluation scenario E (positive sepsis cases follow only implicit labelling strategy) already imply that these models might classify too many cases as positive.

The results for recall at k% and precision at k% confirm this finding. For instance, the model learned with the spy approach and logistic regression for the first and naïve bayes for the second step only detects 35.5% of all true positives in the top-30% of its predictions, and merely 22.4% of its top-30% predictions are true positives (evaluation scenario A). Moreover, precision at k% varies only slightly between the top 10%, 20% and 30% of model predictions. These partial evaluation findings already indicate that this baseline model learned with the spy approach is not potent enough to distinguish between positive and negative examples of sepsis in the unlabeled data. One likely reason is that the logistic regression used in the first step assumes a linear relationship without interactions between cost features and sepsis and therefore is unable to capture non-linear relationships that are essential for effective separation of reliable negative cases. This is confirmed by the cumulative distribution function of model predictions (Panel A of Fig. 1), showing that our baseline model estimates a prediction probability of 1.0 or close to 1.0 for roughly 93% of positive examples, but also for roughly 80% of unlabeled data instances. Finally, the calculation of new sepsis rates (Table 4) reveals that our baseline model would relabel more than 2.1 million cases in the initial dataset, yielding a sepsis rate of more than 82%. This clearly shows that this model is inapt for improving the quality of hospital administrative data for sepsis.

For the model learned with the spy approach and XGBoost for both steps, recall at k% and precision at k% are higher than for the baseline model, implying that XGBoost is more suitable to learn non-linear, complex patterns in the data than a straightforward, less complex classifier such as naïve bayes. While we used default hyperparameters due to the challenges of tuning in a PU learning context, it is nevertheless likely that performance could be further improved with an ideal hyperparameter configuration. Still, when compared to the results for recall at k% and precision at k% of the model learned with AdaSampling, also the second model learned with the spy approach seems inferior. For instance, the model learned with AdaSampling detects all true positive examples in the top 30% of its model predictions, while the model learned with the spy approach and XGBoost only detects 81.4% (recall at 30% of evaluation scenario A). Likewise, 82.7% of the top-10% of model predictions are true positives for the model learned with AdaSampling, yet only 56.9% for the model learned with the spy approach and XGBoost (precision at 10% of evaluation scenario A). In addition, for the latter, with 54.8%, precision at 30% is very close to precision at 10%, showing that the share of true positives among the model’s top predictions does not increase considerably. This indicates that the model also estimates very high prediction probabilities for (too) many unlabeled data instances.

The cumulative distribution function of model predictions confirms this finding (Fig. 1). It reveals that the model learned with the spy approach and XGBoost identifies positive examples almost perfectly, yet at the expense of estimating a prediction probability of 0.0 or close to 0.0 for merely roughly 25% and 1.0 or close to 1.0 for roughly 50% of unlabeled cases (Panel B). On the other hand, Panel C of Fig. 1 shows that the model learned with AdaSampling identifies true positive examples reasonably well, while estimating a prediction probability of 0.0 (1.0) or close to 0.0 (1.0) for roughly 80% (5%) of unlabeled data instances.

As an interim summary, we conclude that the partial evaluation and cumulative distribution functions of model predictions indicate that the baseline model learned with the spy approach seems not to produce meaningful results, while the model learned with the spy approach and XGBoost effectively identifies positive examples, yet seems to discriminate less effectively between positive and negative examples in the unlabeled data than the model learned with AdaSampling.

Finally, the results of the external validity check confirm that the model learned with AdaSampling is the only model that can supply new sepsis rates within a ratio suggested by medical record review studies (cf. Table 4). Accordingly, the plot visualizing the relationship between the new sepsis rates and recalls (Fig. 2) shows that flipping labels of unlabeled data instances part of the top-15% to top-20% of model predictions would yield sepsis rates within the range of empirical ratios between 2.4 and 4.1.

In addition, the plot shows that for the model learned with AdaSampling, recall and the new sepsis rate show a similar relationship as recall and specificity, or precision in (semi-) supervised learning environments: The higher the recall, the higher the number of false positive predictions as indicated by the higher new sepsis rate, pushing the new sepsis rate outside of the range of empirical ratios for recalls higher than roughly 0.75 to 0.80.

One reason why the models learned with the spy approach might be inferior is the relatively low number of reliable negative examples that are identified in the first step. In each cross-validation fold, we use the average of the minimum prediction probability of any spy of 1,000 runs of the first classification step. This yields minimum prediction probabilities between 0.00162 and 0.00215 for our baseline model and 0.00102 to 0.00122 for the model learned with the spy approach and XGBoost (see Table 8 in the supplements).

Using the average of 1,000 runs of the first step is a refinement of the original spy approach study by Liu et al. [31]. In fact, for their empirical application, the authors abandon using spies’ minimum prediction probability estimated in the first step. Instead, they define the minimum probability for reliable negatives $t$ top-down, based on what share of their examples $l$ should be below $t$. The authors experiment with defining $t$ with $l$=5, 10, 15 and 20. The authors’ reasoning for this approach is that among the spies, one outlier’s prediction probability to be a positive example might be 0 or at least too small to robustly identify reliable negatives. We aimed to circumvent this issue in a data-driven way, namely by repeating the estimation of any spy’s minimum prediction probability as part of the first step 1,000 times and averaging results across all runs.

While we appreciate that the authors employed a top-down approach, possibly due to computation constraints at the time of the study more than 20 years ago, we believe setting top-down thresholds is disadvantageous, especially for our aim to showcase a general PU learning application with hospital administrative data. For this purpose, the main disadvantage is that even if thresholds defined top-down yielded more realistic results of the external validity check for our sepsis application, this threshold might have to be adjusted for other PU learning problems with hospital administrative data, e.g., when detecting positive examples of a different underreported diagnosis or procedure.

In addition, results from our baseline model learned with the spy approach might be particularly weak because some of the assumptions necessary for naïve bayes to work well might not hold. Firstly, features might not be independent of one another. For instance, if the operating room cost categories are relatively high, costs for anesthesia will also be relatively high. Likewise, if costs for ICU physician activities are relatively high, ICU overhead costs will also be relatively high. We see this reflected in the descriptive results (Table 1). Secondly, naïve bayes assumes that features are similar in their importance for estimating prediction probabilities. Descriptive results of the cost categories suggest, however, that there are cost categories with distinct differences between positive examples and unlabeled data instances and other cost categories with only little differences. The latter are likely to be less important for model predictions than the former.

Overall, our experimental results show that for our research context and our empirical data, the iterative sampling method AdaSampling combined with radial kernel SVM, a classifier performing well in a high-dimensional feature space and for non-linear relationships, shows the best performance. This is in line with applications in other research fields [35, 36], but unlike others benchmarking AdaSampling with problem-specific algorithms [37].

### Managerial implications

Returning to the relationship between recall and the new sepsis rate (cf. Fig. 2), in our view, choosing the adequate classification threshold depends on the application problem, i.e., the purpose for which the hospital administrative data’s quality needs to be increased. At DRG system level, regulators might want to relabel cases to decrease (unaccounted) cost variation of DRGs. To this end, they need to relabel cases in the unlabeled data with the highest prediction probability and subsequently, reassign them to other, more fitting DRGs according to DRG coding rules. Choosing lower classification thresholds increases the risk of reassigning too many false positives to other DRGs. At the same time, a lower recall associated with higher classification thresholds should be less important from the regulators’ point of view as false negatives are not relabeled. Thus, regulators might rather choose classification thresholds for relabeling unlabeled sepsis cases based on the top 5% or top 10% of model predictions. Considering precision and recall at 10% for evaluation scenarios D and E, regulators might correctly reassign a considerable share of implicit sepsis cases.

Hospitals can use PU learners to identify cases that should be assigned to more complex DRG codes given their cost structure, thereby minimizing negative contribution margins. In a first step, a PU learner could be used to identify sepsis cases in the unlabeled data that are most likely to be mislabeled. In a second step, internal experts (senior physicians and coding specialists) could review the medical records of these patients to see what patients actually had sepsis and thus need to be relabeled. For hospitals, the optimal classification threshold depends on (1) the revenue that could potentially be gained by relabeling unlabeled sepsis cases, i.e., by correctly labeling them, and (2) the costs of medical record review. Considering our results, we believe that an acceptable classification threshold could be chosen based on the top 20% or, for very large hospitals with a higher potential for revenue gain, the top 25% of model predictions. Generally, revenue potential could indeed be substantial, considering the low sensitivity when sepsis cases are identified from raw hospital administrative data [7].

Finally, our findings indicate that PU learning can support research using hospital administrative data, e.g., in health economics, health services research, epidemiology, and disease surveillance. Concretely, we suggest that studies run a sensitivity analysis with relabeled unlabeled sepsis cases. Depending on the specific research question and research area, the classification threshold for relabeling unlabeled sepsis cases should be lower or higher. This is not only relevant for sepsis, but also inpatient complications and patient safety indicators estimated from hospital administrative data. Risk-adjustment using co-morbidities identified from hospital administrative data could also be improved using PU learners.

### Limitations and future research

While we could learn two models with the heuristic, two-step approach “spy” and one model with a robust PU learning approach, it was beyond the scope of this study to conduct data experiments with exemplary models from all four different kinds of PU learning approaches. With our selection, we aimed at including the most common approach in PU learning, i.e., the two-step approach, on the one hand, and a recently increasingly employed approach, i.e., a robust approach, on the other hand. Similarly, for the scope of this study we had to limit ourselves to three different classifiers, namely naïve bayes, XGBoost, and SVM. With this selection, we aimed at balancing the classifiers’ distinct strengths and weaknesses. For instance, XGBoost and SVM are effective in identifying non-linear relationships in high-dimensional spaces while naïve bayes is less complex and less computationally expensive.

The implications of our findings are limited by the fact that we can only partially evaluate our model with traditional evaluation metrics. One possibility to complement model evaluation could be to conduct an empirical Monte Carlo simulation to estimate finite sample performance of PU learners under predefined conditions. Still, given the PU setting, we believe that our model evaluation and especially our external validity check provide sufficient information to assess the effectiveness and potential practical utility of our PU learning models.

Regarding future research, PU learning with hospital administrative data is not limited to sepsis or relabeling other underreported diagnosis codes (e.g., delirium [43]), but could also be a meaningful application for procedure codes for which coding is not mandatory (e.g., emerging technologies such as robotic-assisted surgery [44], given by the CHOP code “00.99.50 Use of a surgical robot”).

Our results further highlight the potential of PU learning for improving the quality of administrative health data in hospital management and healthcare research. Accordingly, as a next step, it could be meaningful to develop a PU learning library for labeling underreported diagnoses and procedures in such datasets. Building on our findings, such a library should focus on robust PU learning models, which demonstrated the best performance in our experiments. The range of classifiers supported within this framework should be expanded, prioritizing those with strong performance on tabular data and in high-dimensional, non-linear settings, for instance ensemble methods such as random forests and gradient boosting. In addition, the library should flexibly incorporate domain knowledge, enabling users to define classification thresholds based on clinical expertise and evidence from the medical literature. In our application, such external information, for example on the prevalence of specific conditions, proved valuable for model evaluation with an external validity check.

## Conclusion

To the best of our knowledge, we are the first to assess whether PU learning is an efficient approach to increase the quality of hospital administrative data, in which only the positive class of diagnosis codes, for instance, is actively labeled. We investigate sepsis, commonly underreported in hospital administrative data, as application example for identifying positive examples in unlabeled data. We hypothesize that hospital cost data at case level could be useful for identifying positive examples of sepsis in unlabeled data as symptoms of sepsis will always need to be treated, incurring costs, even if sepsis as such was not diagnosed, documented, or coded.

Our findings indicate that PU learning can be an effective approach to identify positive examples in unlabeled hospital administrative data in the case of sepsis. At the same time, we find that the quality of model outputs seems to differ substantially between different PU learning approaches and classifiers. Thus, researchers, hospital management, and hospital reimbursement designers should carefully evaluate what combination of approach and classifier works best for their application purpose. We show that for such an assessment, an external validity check is indispensable in the context of applied PU learning with real-world data.

Lastly, relabeling unlabeled examples with a high prediction probability could be a viable approach to increase data quality, potentially improving the performance of certain downstream tasks for DRG system regulators, hospitals, and researchers. Such tasks include DRG coding and derivation of DRG cost weights, hospital revenue and profitability management, disease surveillance, and the estimation of inpatient complication rates or patient safety indicators for outcomes research.

## Supplementary Information

Below is the link to the electronic supplementary material.

Supplementary file1 (PDF 391 KB)

## Data Availability

We use data from the Hospital Case Cost Statistic of the Swiss Federal Statistics Office (*Bundesamt für Statistik - Fallkostenstatistik 2017-2019*). The data is available from the Swiss Federal Statistics Office upon request.

## Footnotes

1 We trained the XGBoost model using the default hyperparameters: n_estimators = 100, learning_rate = 0.3, and max_depth = 6. XGBoost is generally robust to hyperparameter choices and tends to perform well out-of-the-box. Moreover, hyperparameter tuning in a PU learning context is particularly challenging, as the absence of reliably labeled negative instances makes it difficult to evaluate model performance for different hyperparameter configurations.

## Code availability

The code is available for qualified researchers upon request.
