# Supplementary material for: Positive and unlabeled learning from hospital administrative data: a novel approach to identify sepsis cases
Source: Health Care Manag Sci. 2025 Oct 28;28(4):787–805. doi: 10.1007/s10729-025-09733-7 (PMC12743714; doi:10.1007/s10729-025-09733-7)
Supplement: Supplementary file 1 — Supplementary file1 (PDF 391 KB) [file 10729_2025_9733_MOESM1_ESM.pdf]

Supplementary material

Title: Positive and Unlabeled Learning from Hospital Administrative Data: A novel approach for identifying sepsis cases

Journal: Healthcare Management Science

Authors: Vogel, Justus; Cordier, Johannes

Corresponding author: Vogel, Justus; Chair of Health Economics, Policy and Management; School of Medicine; University of St. Gallen; St.-Jakob-Strasse 21, CH-9000 St. Gallen, Switzerland; Justus.Vogel@unisg.ch

Table 5: Explicit and implicit sepsis codes

Code category	Explicit Sepsis-1 coding	Implicit coding (Angus definition)
Explicit sepsis-1 ICD-10-codes	A021; A200; A207; A217; A227; A241; A267; A282; A327; A391; A392; A393; A394; A40; A41; A427; A483; A499; A548; B007; B376; B377; B49; P36; R572; R650; R651	-
ICD-10-codes for infection	-	A00; A01; A02; A03; A04; A05; A06; A07; A08; A09; A15; A16; A17; A18; A19; A20; A21; A22; A23; A24; A25; A26; A27; A28; A32; A36; A37; A38; A39; A40; A41; A42; A43; A44; A46; A48; A49; A50; A54; A55; A56; A59; A65; A690; A691; A692; A698; A699; A74; A75; A77; A78; A79; A80; A81; A83; A84; A85; A86; A87; A88; A89; A90; A91; A92; A93; A94; A95; A96; A97; A98; A99; B00; B01; B02; B03; B04; B05; B06; B07; B08; B09; B25; B26; B27; B33; B34; B37; B38; B39; B40; B41; B42; B43; B44; B45; B46; B47; B48; B49; B50; B51; B52; B53; B54; B55; B58; B60; B64; B67; B95; B96; B97; B98; B99; G00; G01; G02; G03; G04; G05; G06; G07; G08; H050; H602; H700; I32; I33; I38; I39; I40; I41; I80; I981; J01; J02; J03; J04; J05; J06; J09; J10; J11; J12; J13; J14; J15; J16; J17; J18; J20; J21; J22; J36; J390; J391; J440; J441; J85; J86; K35; K36; K37; K5702; K5703; K5712; K5713; K5722; K5723; K5732; K5733; K5742; K5743; K5752; K5753; K5782; K5783; K5792; K5793; K61; K630; K631; K65; K67; K750; K751; K770; K810; L02; L03; L04; L05; L08; M00; M01; M86; N10; N151; N159; N30; N34; N390; N41; N45; N482; N49; N61; N70; N71; N72; N73; N74; N75; N76; N77; N980; O030; O035; O040; O045; O050; O055; O060; O065; O070; O075; O080; O23; O411; O753; O85; O86; O883; O91; O98; P23; P240; P248; P249; P35; P36; P37; P38; P39; P77; P781; R572; R650; R651; T802; T814; T826; T827; T835; T836; T845; T846; T847; T857; T880; U6900; U6940
ICD-10-codes for organ dysfunction	-	D65; D688; D689; D695; D696; E872; F05; G931; G934; I959; J80; J960; J969; J984; K720; K727; K762; K763; N17; N19; R060; R068; R40; R572; R578; R579; R651

Notes: If a code was present for a case, it was counted as a positive example. If the code was not present, the case was counted as a negative or rather unlabeled example. For the Angus definition, both at least one code from the ICD-10-codes for infection and at least one code from the ICD-10-codes for organ dysfunction needed to be present for a case to be counted as a positive example. We considered both primary and secondary diagnoses. If an ICD-10-code is listed with only two or three characters, this means that all subordinate codes (i.e., all more detailed ICD-10-codes with three of four characters) were considered (if available for the particular ICD-10-code). Definitions are based on [1] and [2].

Table 6: Included attributes PU data classifier could learn from

Category	Attribute
Hospital costs at case level	Total costs
	Pharmaceuticals
	Blood and blood products
	Medical material
	Implants
	Medical, diagnostic, and therapeutic third-party services (excl. doctors' fees)
	Doctor's fees (not subject to social security contributions)
	Medical fees, hospital doctors (subject to social security contributions)
	Medical fees, attending physicians (subject to social security contributions)
	Patient transportation by third parties
	Other patient-related third-party services
	Other expenses for patients
	Patient administration, overheads excl. IUC
	Patient administration, IUC
	Operating room, overheads excl. IUC
	Operating room, IUC
	Operating room doctors - activities 6a, overheads excl. IUC
	Operating room doctors - activities 6a, IUC
	Anesthesia, overheads excl. IUC
	Anesthesia, IUC
	Intensive care unit, overheads excl. IUC
	Intensive care unit, IUC
	Intensive care unit physicians - activities 6b1, overheads excl. IUC
	Intensive care unit physicians - activities 6b1, IUC
	Intermediate Care Units (IMCU), overheads excl. IUC
	Intermediate Care Units (IMCU), IUC
	IMCU physicians - activities 6b2, overheads excl. IUC
	IMCU physicians - activities 6b2, IUC
	Emergency, overheads excl. IUC
	Emergency, IUC
	Emergency physician services - activities 6b3, overheads excl. IUC
	Emergency physician services - activities 6b3, IUC
	Imaging procedures, overheads excl. IUC
	Imaging procedures, IUC
	Delivery room, overheads excl. IUC
	Delivery room, IUC
	Delivery room doctors - activities 6b4, overheads excl. IUC
	Delivery room doctors - activities 6b4, IUC
	Nuclear medicine and radiation oncology, overheads excl. IUC
	Nuclear medicine and radiation oncology, IUC
	Laboratory, overheads excl. IUC
	Laboratory, IUC
	Dialysis, overheads excl. IUC
	Dialysis, IUC
	Physicians, activities 1-5, overheads excl. IUC
	Physicians, activities 1-5, IUC
	Physiotherapy, overheads excl. IUC
	Physiotherapy, IUC
	Occupational therapy, overheads excl. IUC
	Occupational therapy, IUC
	Speech therapy, overheads excl. IUC

Category	Attribute
	Speech therapy, IUC
	Non-medical therapies and consultations, overheads excl. IUC
	Non-medical therapies and consultations, IUC
	Medical and therapeutic diagnostics, overheads excl. IUC
	Medical and therapeutic diagnostics, IUC
	Medical and therapeutic diagnostics doctors - activities 6b5, overheads excl.
	Medical and therapeutic diagnostics - activities 6b5, IUC
	Nursing, overheads excl. IUC
	Nursing care, IUC
	Hotel rooms, overheads excl. IUC
	Hotel rooms, IUC
	Hotel kitchen, overheads excl. IUC
	Hotel kitchen, IUC
	Hotel service, overheads excl. IUC
	Hotel service, IUC
	Other service providers, overheads excl. IUC
	Other service providers, IUC
	Pathology, overheads excl. IUC
	Pathology, IUC
	Rescue and ambulance service (secondary transport only), overheads excl. IUC
	Rescue and ambulance service (secondary transports only), IUC
Aggregated medical information	ICD chapter of main diagnosis
	Number of procedures per CHOP chapter

Annotations: IUC = Infrastructure Usage Costs; ICD = International Classification of Diseases; CHOP = Swiss Operation and Procedure Catalogue. All attributes of the category hospital costs at case level are in Swiss francs and continuous variables with a theoretical range between 0 and positive infinity. ICD chapters are dummy variables. The number of procedures per CHOP chapter are natural numbers including 0. Overall, there are 23 ICD (21 relevant for main diagnoses) and 19 CHOP chapters.

Fig 3 Constellations for counting positive examples in the evaluation scenarios (Venn diagram)

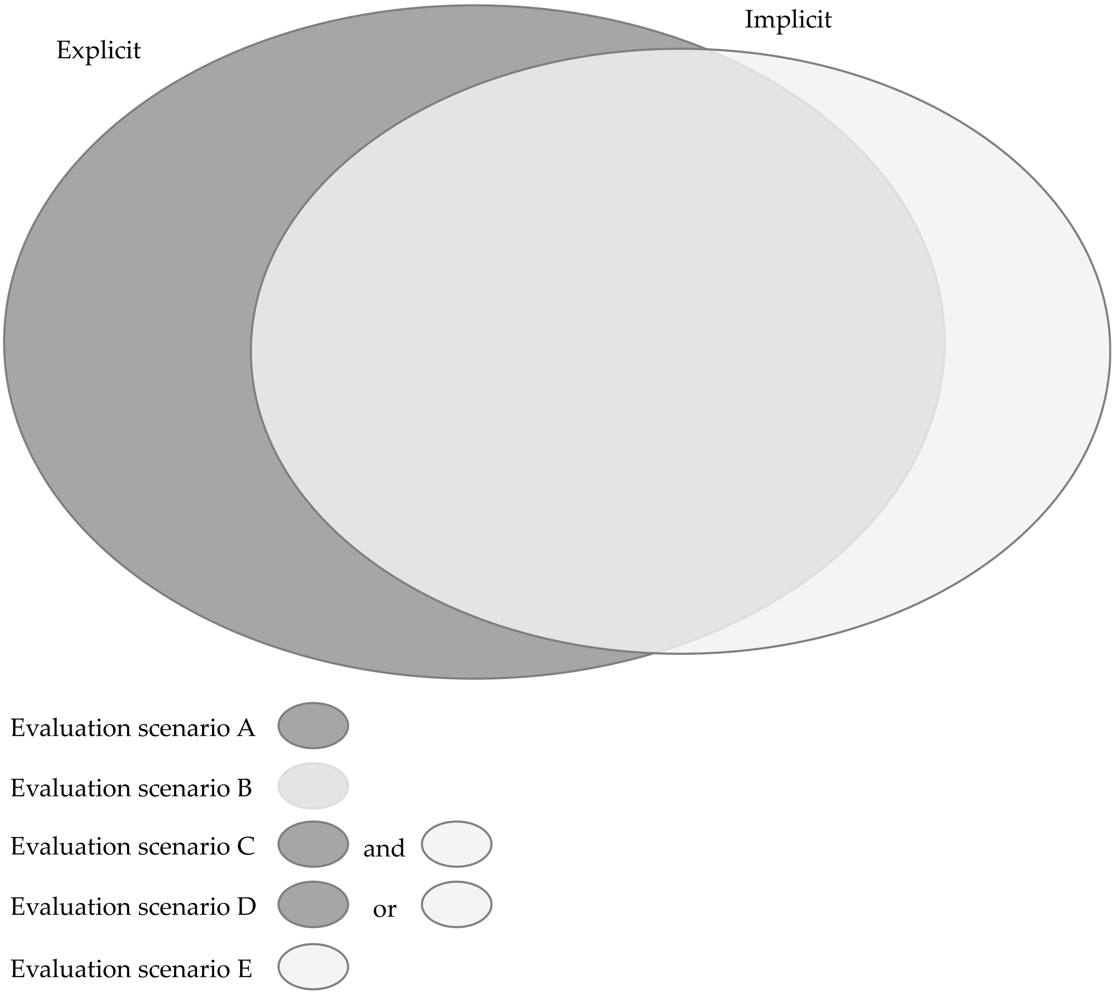

Table 7: Full descriptive results

Attribute	Total sample (n=313,433)		Labeled sepsis cases (n=63,434)		Unlabeled cases (n=250,000)	
	Mean (SD)	Median (25th – 75th)	Mean (SD)	Median (25th – 75th)	Mean (SD)	Median (25th – 75th)
Total costs	17,260 (37,739)	8,537 (4,953-16,319)	37,098 (72,376)	15,186 (8,013-35,426)	12,226 (18,201)	7,626 (4,464-13,665)
Variable direct costs [CHF]						
Pharmaceuticals	479 (2,938)	87 (20-242)	1,428 (5,846)	271 (100-841)	238 (1,366)	67 (12-173)
Blood and blood products	204 (2,846)	0 (0-0)	779 (6,003)	0 (0-0)	58 (953)	0 (0-0)
Medical material	576 (1,974)	81 (1-428)	1,029 (3,484)	113 (18-615)	461 (1,318)	73 (0-397)
Implants	454 (2,659)	0 (0-0)	394 (3,654)	0 (0-0)	469 (2,340)	0 (0-0)
Medical, diagnostic, and therapeutic third-party services (excl. doctors' fees)	241 (1,093)	0 (0-120)	607 (1,962)	16 (0-490)	148 (693)	0 (0-71)
Doctor's fees (not subject to social security contributions)	271 (1,282)	0 (0-0)	278 (2,211)	0 (0-0)	270 (906)	0 (0-0)
Medical fees, hospital doctors (subject to social security contributions)	22 (195)	0 (0-0)	32 (261)	0 (0-0)	20 (174)	0 (0-0)
Medical fees, attending physicians (subject to social security contributions)	17 (176)	0 (0-0)	21 (208)	0 (0-0)	16 (167)	0 (0-0)
Patient transportation by third parties	53 (343)	0 (0-0)	117 (581)	0 (0-0)	36 (246)	0 (0-0)
Other patient-related third-party services	15 (532)	0 (0-0)	35 (674)	0 (0-0)	10 (490)	0 (0-0)
Other expenses for patients	11 (193)	0 (0-0)	26 (336)	0 (0-0)	7 (134)	0 (0-0)
Fixed indirect costs [CHF]						
Patient administration, overheads excl. IUC	197 (192)	172 (112-241)	213 (206)	185 (120-246)	193 (189)	168 (112-238)
Patient administration, IUC	17 (23)	11 (6-21)	19 (29)	11 (7-21)	17 (21)	11 (6-21)
Operating room, overheads excl. IUC	714 (2106)	0 (0-886)	1003 (3806)	0 (0-34)	641 (1364)	0 (0-964)
Operating room, IUC	183 (655)	0 (0-212)	238 (842)	0 (0-2)	170 (597)	0 (0-235)
Operating room doctors - activities 6a, overheads excl. IUC	237 (873)	0 (0-8)	349 (1402)	0 (0-0)	209 (673)	0 (0-57)
Operating room doctors - activities 6a, IUC	13 (68)	0 (0-0)	19 (108)	0 (0-0)	11 (54)	0 (0-0)
Anesthesia, overheads excl. IUC	601 (1565)	0 (0-790)	867 (2788)	0 (0-514)	533 (1037)	0 (0-825)
Anesthesia, IUC	58 (152)	0 (0-72)	81 (266)	0 (0-42)	52 (104)	0 (0-76)
Intensive care unit, overheads excl. IUC	1,919 (13,374)	0 (0-0)	7,763 (27,900)	0 (0-3,495)	437 (3,986)	0 (0-0)
Intensive care unit, IUC	181 (1251)	0 (0-0)	730 (2601)	0 (0-293)	42 (386)	0 (0-0)
Intensive care unit physicians - activities 6b1, overheads excl. IUC	365 (3,003)	0 (0-0)	1,448 (6,294)	0 (0-181)	90 (940)	0 (0-0)
Intensive care unit physicians - activities 6b1, IUC	15 (149)	0 (0-0)	57 (314)	0 (0-0)	4 (49)	0 (0-0)
IMCU, overheads excl. IUC	269 (2,844)	0 (0-0)	818 (5,735)	0 (0-0)	129 (1,304)	0 (0-0)
Intermediate Care Units (IMCU), IUC	19 (231)	0 (0-0)	57 (461)	0 (0-0)	10 (112)	0 (0-0)

Attribute	Total sample (n=313,433)		Labeled sepsis cases (n=63,434)		Unlabeled cases (n=250,000)	
	Mean (SD)	Median (25th – 75th)	Mean (SD)	Median (25th – 75th)	Mean (SD)	Median (25th – 75th)
IMCU physicians - activities 6b2, overheads excl. IUC	38 (431)	0 (0-0)	110 (812)	0 (0-0)	20 (254)	0 (0-0)
IMCU physicians - activities 6b2, IUC	2 (34)	0 (0-0)	5 (46)	0 (0-0)	1 (31)	0 (0-0)
Emergency, overheads excl. IUC	185 (323)	0 (0-307)	351 (370)	306 (85-478)	143 (296)	0 (0-231)
Emergency, IUC	19 (36)	0 (0-30)	36 (41)	26 (6-53)	15 (34)	0 (0-21)
Emergency medical services - activities 6b3, overheads excl. IUC	89 (186)	0 (0-117)	166 (227)	67 (0-279)	69 (168)	0 (0-41)
Emergency medical services - activities 6b3, IUC	4 (12)	0 (0-1)	7 (15)	0 (0-7)	3 (10)	0 (0-0)
Imaging procedures, overheads excl. IUC	277 (697)	41 (0-291)	668 (1190)	305 (87-758)	178 (448)	0 (0-179)
Imaging procedures, IUC	77 (201)	8 (0-76)	182 (331)	77 (24-203)	50 (140)	0 (0-47)
Delivery room, overheads excl. IUC	117 (640)	0 (0-0)	7 (197)	0 (0-0)	145 (707)	0 (0-0)
Delivery room, IUC	16 (99)	0 (0-0)	1 (28)	0 (0-0)	20 (110)	0 (0-0)
Delivery room doctors - activities 6b4, overheads excl. IUC	19 (156)	0 (0-0)	2 (62)	0 (0-0)	24 (172)	0 (0-0)
Delivery room doctors - activities 6b4, IUC	1 (12)	0 (0-0)	0 (7)	0 (0-0)	1 (13)	0 (0-0)
Nuclear medicine and radiation oncology, overheads excl. IUC	22 (314)	0 (0-0)	34 (375)	0 (0-0)	19 (296)	0 (0-0)
Nuclear medicine and radiation oncology, IUC	8 (116)	0 (0-0)	13 (138)	0 (0-0)	7 (110)	0 (0-0)
Laboratory, overheads excl. IUC	476 (1755)	100 (0-371)	1,505 (3,494)	584 (260-1303)	215 (652)	60 (0-214)
Laboratory, IUC	56 (217)	10 (0-40)	178 (432)	62 (24-151)	25 (84)	6 (0-23)
Dialysis, overheads excl. IUC	60 (1020)	0 (0-0)	244 (2161)	0 (0-0)	13 (330)	0 (0-0)
Dialysis, IUC	7 (115)	0 (0-0)	26 (241)	0 (0-0)	2 (42)	0 (0-0)
Physicians, activities 1-5, overheads excl. IUC	1,378 (3,084)	783 (223-1,579)	2,614 (5,393)	1,481 (785-2,779)	1,065 (2,014)	647 (131-1,315)
Physicians, activities 1-5, IUC	95 (229)	37 (2-103)	173 (363)	78 (26-179)	75 (175)	29 (0-87)
Physiotherapy, overheads excl. IUC	255 (1,096)	0 (0-200)	623 (1,845)	196 (0-615)	161 (774)	0 (0-124)
Physiotherapy, IUC	34 (202)	0 (0-24)	74 (192)	21 (0-76)	23 (203)	0 (0-14)
Occupational therapy, overheads excl. IUC	43 (413)	0 (0-0)	64 (561)	0 (0-0)	38 (366)	0 (0-0)
Occupational therapy, IUC	5 (62)	0 (0-0)	7 (70)	0 (0-0)	5 (59)	0 (0-0)
Speech therapy, overheads excl. IUC	17 (240)	0 (0-0)	44 (403)	0 (0-0)	11 (176)	0 (0-0)
Speech therapy, IUC	2 (23)	0 (0-0)	4 (41)	0 (0-0)	1 (15)	0 (0-0)
Non-medical therapies and consultations, overheads excl. IUC	145 (825)	0 (0-28)	263 (821)	8 (0-237)	115 (824)	0 (0-16)
Non-medical therapies and consultations, IUC	15 (97)	0 (0-3)	27 (98)	1 (0-21)	12 (97)	0 (0-1)
Medical and therapeutic diagnostics, overheads excl. IUC	132 (637)	0 (0-0)	239 (832)	0 (0-128)	105 (574)	0 (0-0)
Medical and therapeutic diagnostics, IUC	36 (220)	0 (0-0)	65 (214)	0 (0-33)	29 (221)	0 (0-0)

Attribute	Total sample (n=313,433)		Labeled sepsis cases (n=63,434)		Unlabeled cases (n=250,000)	
	Mean (SD)	Median (25th – 75th)	Mean (SD)	Median (25th – 75th)	Mean (SD)	Median (25th – 75th)
Medical and therapeutic diagnostics doctors – activ. 6b5, overheads excl. IUC	68 (379)	0 (0-0)	158 (691)	0 (0-17)	45 (237)	0 (0-0)
Medical and therapeutic diagnostics doctors - activities 6b5, IUC	3 (21)	0 (0-0)	7 (34)	0 (0-0)	2 (16)	0 (0-0)
Nursing care, overheads excl. IUC	3,606 (7,742)	1,577 (760-3,590)	7,322 (12,556)	3,801 (1,741-8,210)	2,663 (5,546)	1,328 (681-2,717)
Nursing care, IUC	251 (604)	104 (39-247)	473 (954)	217 (87-511)	194 (459)	88 (35-199)
Hotel rooms, overheads excl. IUC	361 (742)	183 (86-381)	685 (1,245)	367 (188-736)	279 (514)	154 (74-306)
Hotel rooms, IUC	261 (618)	120 (35-274)	483 (1,036)	235 (82-526)	205 (436)	102 (27-227)
Hotel kitchen, overheads excl. IUC	444 (730)	247 (114-510)	793 (998)	511 (273-965)	356 (613)	206 (97-404)
Hotel kitchen, IUC	62 (119)	30 (10-69)	106 (158)	60 (24-127)	51 (104)	26 (8-57)
Hotel service, overheads excl. IUC	94 (309)	0 (0-73)	156 (451)	0 (0-126)	78 (258)	0 (0-61)
Hotel service, IUC	7 (32)	0 (0-4)	10 (32)	0 (0-6)	6 (32)	0 (0-3)
Other service providers, overheads excl. IUC	39 (201)	0 (0-22)	75 (279)	4 (0-53)	30 (174)	0 (0-17)
Other service providers, IUC	12 (91)	0 (0-3)	26 (157)	0 (0-8)	9 (64)	0 (0-2)
Pathology, overheads excl. IUC	43 (290)	0 (0-0)	78 (436)	0 (0-0)	35 (239)	0 (0-0)
Pathology, IUC	8 (59)	0 (0-0)	15 (94)	0 (0-0)	6 (46)	0 (0-0)
Rescue/ ambulance service (secondary transport), overheads excl. IUC	10 (116)	0 (0-0)	25 (186)	0 (0-0)	6 (89)	0 (0-0)
Rescue/ ambulance service (secondary transport), IUC	1 (17)	0 (0-0)	3 (24)	0 (0-0)	1 (15)	0 (0-0)
CHOP chapters: Surgeries of the...						
Nervous system	0.09 (0.45)	0 (0-0)	0.08 (0.59)	0 (0-0)	0.09 (0.41)	0 (0-0)
Endocrine system	0 (0.07)	0 (0-0)	0 (0.05)	0 (0-0)	0 (0.08)	0 (0-0)
Eye	0.02 (0.25)	0 (0-0)	0 (0.07)	0 (0-0)	0.02 (0.27)	0 (0-0)
Ears	0.01 (0.11)	0 (0-0)	0 (0.07)	0 (0-0)	0.01 (0.11)	0 (0-0)
Nose, mouth, throat	0.04 (0.32)	0 (0-0)	0.02 (0.24)	0 (0-0)	0.04 (0.33)	0 (0-0)
Respiratory system	0.07 (0.52)	0 (0-0)	0.23 (0.96)	0 (0-0)	0.04 (0.31)	0 (0-0)
Cardiovascular system	0.29 (1.55)	0 (0-0)	0.68 (2.85)	0 (0-0)	0.19 (0.95)	0 (0-0)
Hematopoietic and lymphatic system	0.02 (0.19)	0 (0-0)	0.04 (0.26)	0 (0-0)	0.02 (0.17)	0 (0-0)
Digestive tract	0.28 (1.19)	0 (0-0)	0.68 (2.24)	0 (0-0)	0.18 (0.67)	0 (0-0)
Urinary organs	0.1 (0.46)	0 (0-0)	0.22 (0.68)	0 (0-0)	0.07 (0.37)	0 (0-0)
Male sexual organs	0.01 (0.15)	0 (0-0)	0.01 (0.16)	0 (0-0)	0.02 (0.14)	0 (0-0)
Female sexual organs	0.05 (0.33)	0 (0-0)	0.01 (0.16)	0 (0-0)	0.05 (0.36)	0 (0-0)
Obstetric procedures	0.11 (0.5)	0 (0-0)	0 (0.08)	0 (0-0)	0.14 (0.56)	0 (0-0)
Musculoskeletal system	0.29 (1.05)	0 (0-0)	0.22 (1.24)	0 (0-0)	0.31 (0.99)	0 (0-0)
Integumentary system	0.14 (1.14)	0 (0-0)	0.38 (2.2)	0 (0-0)	0.08 (0.62)	0 (0-0)
Other diagn. or therapeutic procedures	2.44 (5.67)	1 (0-2)	3.21 (4.78)	2 (0-4)	2.24 (5.86)	0 (0-1)

Attribute	Total sample (n=313,433)		Labeled sepsis cases (n=63,434)		Unlabeled cases (n=250,000)	
	Mean (SD)	Median (25th – 75th)	Mean (SD)	Median (25th – 75th)	Mean (SD)	Median (25th – 75th)
Measurement instruments	0.84 (5.97)	0 (0-0)	0.66 (5.44)	0 (0-0)	0.88 (6.1)	0 (0-0)
Rehabilitation	0.02 (0.14)	0 (0-0)	0.01 (0.11)	0 (0-0)	0.02 (0.15)	0 (0-0)
Procedures not classified elsewhere	0.25 (0.77)	0 (0-0)	0.31 (0.95)	0 (0-0)	0.23 (0.71)	0 (0-0)
Sample descriptives not used for classification (Age, gender, Elixhauser Comorbidity Index Score), and additional descriptive information (mortality, positive class label mean)						
Age	55.1 (27.1)	60 (35-75)	69.3 (20.3)	75 (60-85)	51.5 (27.4)	55 (30-75)
Share of female patients	49.19%		57.52%		47.07%	
Elixhauser Comorbidity Index Score	4.9		11.8		3.2	
Number of unweighted Elixhauser diagnoses	2.0		3.9		1.5	
Inpatient mortality rate	3.89%		13.99%		1.33%	
Positive class label mean	0.2024					

Annotations: CHOP = Swiss Operation and Procedure Catalogue; IMCU = Intermediate Care Units; IUC = Infrastructure Usage Costs. All costs are in Swiss francs and rounded to full numbers. All shares, percentages, and CHOP chapters are rounded to two decimals. Age is rounded to one decimal. Labeled sepsis cases follow the explicit coding definition presented in Table 5 above. We calculated the Elixhauser Comorbidity Index Score according to Van Walraven et al. [3].The inpatient mortality rate is not risk-adjusted.
Source: Hospital Case Cost Dataset from the Swiss Federal Statistics Office, data years 2017 to 2019.

Table 8: Overview of train sets of the cross validation folds – spy approach

CV fold	Positive class label mean	Number of positive examples	Number of reliable negative examples	Minimum prediction probability of any spy
Model learned with "spy" approach, logistic regression for first and naïve bayes classifier for second step				
1	0.8061	50,744	12,206	0.00211
2	0.8512	50,772	8,879	0.00210
3	0.8541	50,722	8,667	0.00162
4	0.8022	50,669	12,495	0.00214
5	0.8044	50,831	12,363	0.00215
Model learned with "spy" approach, XGBoost for both steps				
1	0.59613	50,744	34,378	0.00106
2	0.61367	50,772	31,963	0.00105
3	0.60727	50,722	32,803	0.00102
4	0.58749	50,669	35,577	0.00115
5	0.56738	50,831	38,758	0.00122

Annotations: CV = cross-validation. Positive class label means are rounded to five decimals. Note that positive class label means between the two models only differ starting at the five decimal. The column "Minimum prediction probabilities of any spy" shows the average of the 1,000 runs per cross-validation fold.

Table 9: Overview of train sets of the cross validation folds – AdaSampling approach

CV fold	Positive class label mean	Number of positive examples	Number of negative (or rather unlabeled) examples
1	0.2024	50,744	200,004
2	0.2025	50,772	199,976
3	0.2023	50,722	200,026
4	0.2021	50,669	200,079
5	0.2027	50,831	199,917

Annotations: CV = cross-validation. Positive class label means are rounded to four decimals.

Bibliography

1. Schwarzkopf D, Rose N, Fleischmann-Struzek C, et al (2023) Understanding the biases to sepsis surveillance and quality assurance caused by inaccurate coding in administrative health data. Infection 1:1–15. https://doi.org/10.1007/s15010-023-02091-y
2. Angus DC, Linde-Zwirble WT, Lidicker J, et al (2001) Epidemiology of severe sepsis in the United States: analysis of incidence, outcome, and associated costs of care. Crit Care Med 29:1303–1310. https://doi.org/10.1097/00003246-200107000-00002
3. Van Walraven C, Austin PC, Jennings A, et al (2009) A modification of the Elixhauser comorbidity measures into a point system for hospital death using administrative data. Med Care 47:626–633. https://doi.org/10.1097/MLR.0B013E31819432E5
